# The impact of insertion bias into piRNA clusters on the invasion of transposable elements

**DOI:** 10.1186/s12915-025-02370-0

**Published:** 2025-08-15

**Authors:** Shashank Pritam, Almorò Scarpa, Robert Kofler, Sarah Signor

**Affiliations:** 1 North Dakota State University, Fargo, ND USA; 2 Institut für Populationsgenetik, Vetmeduni Vienna, Veterinärplatz 1, 1210 Wien, Austria; 3 Vienna Graduate School of Population Genetics, Vetmeduni Vienna, Vienna, Austria

**Keywords:** Insertion bias, Forward simulations, PiRNA cluster, Transposable elements, TE-host interactions, Population genetics

## Abstract

**Background:**

In our current understanding of transposable element (TE) invasions, TEs move freely until they accidentally insert into a piRNA cluster, where they are silenced by the production of piRNA cognate to the TE. Under this model, one would expect that selection might favor TEs that avoid piRNA clusters. However, empirical observations show that some TEs, such as the *P*-element, insert into piRNA clusters preferentially. We were thus wondering if such a bias, by minimizing harm to the host, could facilitate the spread of TEs throughout a population.

**Results:**

We performed extensive forward simulations of TE invasions with different insertion biases into piRNA clusters to determine if there was ever a situation in which the insertion bias was beneficial to the TE. We found that insertion bias significantly altered the invasion dynamics of TEs, primarily by changing the number of TE copies in individuals before silencing. Insertion into a piRNA cluster reduced the deleterious effects of TEs to the host population, but we found that TEs avoiding piRNA clusters out-compete TEs with a bias toward cluster insertions. Insertion bias was only beneficial to the TE when there was negative selection against TEs and a lack of recombination.

**Conclusions:**

Different TEs show different insertion biases into piRNA clusters suggesting they are an attribute of the TE not the host, yet scenarios in which this is beneficial for TE propagation are quite limited. This opens up an interesting area for future research into the dynamics of insertion bias during TE invasions.

**Supplementary information:**

The online version contains supplementary material available at 10.1186/s12915-025-02370-0.

## Background

Transposable elements (TEs) are selfish genetic elements that replicate within host genomes. TEs can make up a substantial fraction of eukaryotic genomes—from as low as 3% in pufferfish [1] to 20% in *Drosophila* and up to 90% in maize [2–4]. TEs cause genomic instability through various mechanisms including double-stranded DNA breaks, ectopic recombination, and disruption of coding sequences [5]. Notably, TE proliferation drives major genome size variation, for example, in *D. melanogaster* TEs have increased the genome size by 1 MB in the last 200 years [6, 7]. Given that the majority of TE insertions are deleterious, it was previously hypothesized that TE copy number is maintained through a balance between transposition and negative selection [8–10]. However, this supposition has been challenged by the discovery of a dedicated system for the suppression of TEs.

In the model organism *Drosophila*, for example, it has been demonstrated that TEs are actively suppressed by a dedicated small RNA pathway [11]. In this system, small RNAs termed *piwi*-interacting RNAs (piRNAs) are produced by TE-rich genomic regions called piRNA clusters and guide Argonaute proteins to silence TEs pre- and post-transcriptionally [12, 13]. These TE-rich genomic regions which produce piRNA are discrete and are referred to as piRNA clusters. piRNA clusters are generally found in heterochromatin, near the euchromatic boundary [11]. They make up a substantial proportion of the genome, for example, in *D. melanogaster* piRNA clusters are 3.5% of the total genome. They are TE dense regions that contain recently active full-length insertions to small degraded fragments of older invasions. Several studies have found that a single insertion of a TE into a cluster region was sufficient to initiate silencing of a TE [14–16].

The observation that a single TE insertion into a piRNA cluster silenced the TE led to the development of the “trap model” of TE suppression—under this model an invading TE jumps into a piRNA cluster, which triggers the emergence of piRNAs complementary to the TE [16–21]. This suppression prevents the TE from any further transposition. According to the trap model, three key expectations must hold—piRNAs should be produced from sequences inserted in piRNA clusters, insertion into a piRNA cluster should be sufficient to suppress a TE, and TEs should not be present in many copies within piRNA clusters [15–20]. Simulations of TEs invasions under the trap model have revealed additional expectations that can be empirically tested. For example, these simulations have established that TEs are initially silenced by segregating cluster insertions, and that around four cluster insertions in a population are necessary to stop a TE invasion [6, 22, 23]. TE invasions proceed through three stages—rapid, where the TE is proliferating uncontrolled in the host genome, shotgun, where cluster insertions are segregating in the population but remain unfixed, and inactive [6, 23]. Existing work largely meets these expectations and supports the trap model of TE suppression [11, 15, 16, 24–28].

However, there are some observations that do not fit with the expectations of the trap model. For example, there are fewer cluster insertions than expected [29–32]. In addition, deleting three large piRNA clusters in *D. melanogaster* had *little detectable* effect on fertility or TE transcript levels [33]. The trap model also assumes random TE insertion *for most TE families*; however, there is some evidence that TEs insert preferentially into piRNA-producing regions. For example, [34] found that piRNA clusters must constitute about 0.2–3% of the genome to effectively suppress transposons. Yet, some species (e.g., human and mouse) have piRNA clusters that do not meet this minimum size, without suffering the consequences of uncontrolled TE transposition. An insertion bias into piRNA clusters could compensate for small piRNA clusters. In fact, some TEs do show evidence of insertion bias, such as the *P*-element which inserts preferentially into a piRNA cluster called *X-TAS* [22, 23]. Investigations of novel insertions revealed that several TE families could have an insertion bias toward piRNA clusters [35, 36]. A high rate of new *Gypsy*-family insertions was also observed for *flamenco*, a somatic piRNA cluster [16]. An insertion bias into piRNA clusters may be an evolutionary adaptation. Such a bias could allow the TE to accumulate a sufficient number of TE copies in an organism to ensure efficient transmission to the next generation, yet prevent the accumulation of excessive copies that could harm the host. Alternatively, an insertion bias into piRNA clusters could be the by-product of an insertion bias into different regions showing some shared properties with piRNA clusters. For example, it has been suggested that TEs may have a preference for safe havens, where insertions have a low impact on host fitness, such as heterochromatin. An insertion bias into heterochromatin could, as a byproduct, also lead to an over-representation of insertions in piRNA clusters which are typically at the boundary between heterochromatin and euchromatin.

Previous work investigated whether TEs may evolve self-regulation by reducing the transposition rates to minimize host damage [8]. A reduced transposition rate could solely evolve under a few scenarios, such as low recombination rates. Thus, we aimed to investigate the effect of an insertion bias into piRNA clusters on the invasion dynamics of TEs and to test whether such an insertion bias could be an adaptive trait. To investigate the possibility that TEs have an insertion bias into piRNA clusters, we wanted to simulate different scenarios in which an insertion bias could potentially be beneficial to the TE. The goal of the present work is to determine with simulations whether insertion bias could be adaptive. We simulated TE invasions under varying bias strengths using invadego [29]. Our results demonstrate that insertion bias into piRNA clusters generally does not benefit TEs, challenging the hypothesis of evolved strategic insertion.

## Results

### Model implementation and assumptions

In the absence of insertion bias, the probability that a TE will insert into a piRNA cluster is determined by the amount of the genome that the piRNA cluster occupies. For example, if piRNA clusters make up 3% of the genome, then that is also the baseline probability of a cluster insertion. When a TE shows positive insertion bias, its probability of inserting into a clusters would exceed 3%, leading to more insertions in piRNA clusters than expected by chance.

**Fig. 1:**
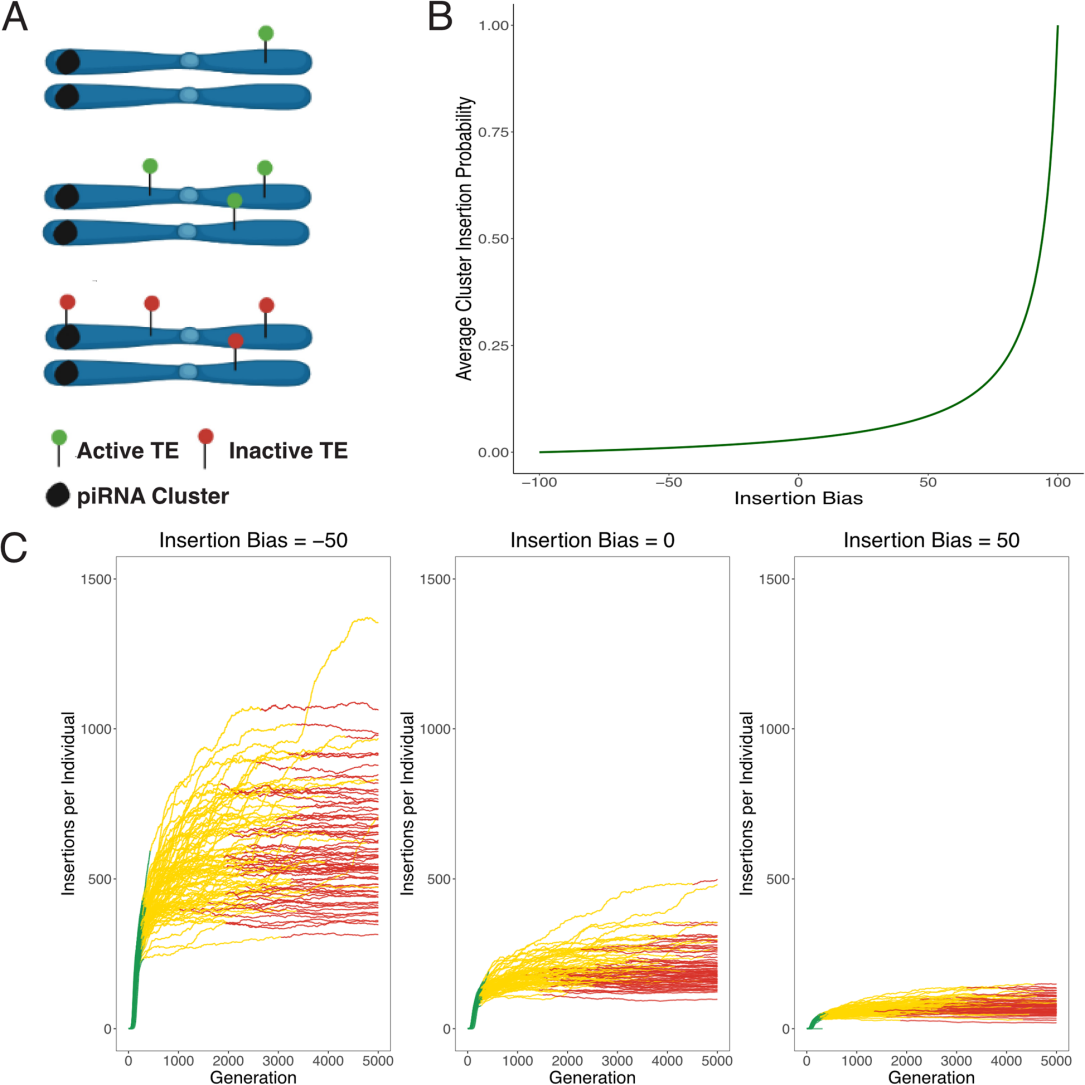
TE invasion modeling. **A** A simple overview of our model assumptions. We begin the simulation with TE insertions in the population to avoid loss due to drift. The TE increases in copy number until it is inserted into a piRNA cluster and is silenced. **B** Relationship between insertion bias and probability of TE integration within piRNA cluster. **C** Effect of insertion bias on TE abundance during three invasion phases (color-coded)—rapid, shotgun, and inactive. Higher bias into cluster regions correlates with reduced TE accumulation within the genome. Each line represents one of 100 simulations

In our simulations, an insertion bias is a characteristic of the TE, not the host. We assume that a TE is active in all individuals which do not have an insertion in a piRNA cluster (Fig. 1A). This assumption aligns with the “trap model” proposed in previous studies, where the proliferation of an active TE is halted when one copy inserts into a piRNA cluster, subsequently deactivating all TE copies in *trans* [16–21].

While the piRNA pathway is broadly conserved, the specific parameters of the “trap model” are based on the well-characterized dual-stranded germline clusters of *Drosophila* [18]. In this system, TE insertions can occur in any orientation within a cluster and still generate piRNAs that effectively silence corresponding TEs. This model is further supported by findings that individual euchromatic TE insertions can trigger the formation of novel dual-stranded piRNA clusters in *Drosophila*, which contributes to a more effective defense against TE expansion. The dual-stranded nature of these clusters is crucial for their function, as it allows for the production of both sense and antisense piRNAs, boosting the silencing capacity against active TEs [37]. It is also important to note that the size and distribution of piRNA clusters play a significant role in their effectiveness against TE invasions.

In the context of our simulations, these findings highlight the complexity of TE-piRNA cluster interactions and the importance of considering factors such as insertion bias, cluster size, and spatial organization when modeling the dynamics of TE invasions and their suppression by piRNA clusters. In the first set of simulations performed here, TE insertions are assumed to be selectively neutral. There were two reasons for the approach. First, we are investigating the behavior of a complex system and the simplest possible scenario should be initially explored before adding additional complicating factors. Second, the fitness effect of many TE insertions is also controversial—while it is unlikely that a system such as the piRNA pathway would have evolved without a negative fitness effect of TEs, there is ambiguous evidence that individual TE insertions are necessarily deleterious [38–40]. For example, we expect the X chromosome to have fewer TE insertions than the autosomes if they are negatively selected because the X chromosome is directly exposed to selection in males. However, in *Drosophila* the X chromosome does not show different patterns of TE insertions relative to the autosomes [41, 42]. Furthermore, ectopic recombination could be the source of negative fitness effects from TE insertions, but there is not strong evidence of a relationship between recombination rate and TE density outside of *Drosophila* [43–45].

Empirical work on TE invasions supports an alternative scenario, where TE invasions are halted by many segregating cluster insertions [22]. Other empirical work on the *P*-element also supports this scenario, where the invasion of the TE plateaued at around 20 generations, during which all observed cluster insertions were segregating at low frequency [30].

The following parameters were used as default for all simulations unless otherwise specified: a transposition rate of 0.1, a population size of *N *= 1000, and piRNA clusters of 300 kb (3%) of the genome. We also used five chromosome arms of 10 Mbp each and a recombination rate of 4 cM/Mbp. An important base parameter is a starting population of 100 randomly inserted TEs in the population of 1000. These insertions will have a population frequency of $\textit{f} = 1 / (2 * 1,000)$. Triggering a TE invasion with multiple insertions avoids early loss of TEs due to stochastic genetic drift [23, 29]. For every simulation, we performed 100 replicates. We initially simulated TE insertions with no negative selection, but later incorporated scenarios with selection against TE insertions.

### Effect of insertion bias on TE invasions

Here we hypothesized that an insertion bias may be beneficial to the TE as it minimizes damage to the host while still enabling the TE to spread to appreciable copy numbers. We tested this with extensive forward simulations under the trap model, which assumes that a TE is spreading in a population until one copy jumps into a piRNA cluster (Fig. 1A). An insertion in a piRNA cluster silences all copies of the TE. We modeled an insertion bias with values between −100 (complete avoidance of piRNA cluster) and +100 (all insertions in piRNA clusters), which is translated into an insertion probability (as detailed in the Methods section). A value of 0 indicates the absence of any insertion bias. The insertion bias can be translated into the probability that a TE jumps into a piRNA cluster (see Fig. 1B). Note that in an unbiased case ($bias=0$) the probability of inserting into a piRNA cluster corresponds to the genomic proportion of the piRNA cluster (i.e., 0.03 in our simulations).

We first tested whether an insertion bias has an effect on the invasion dynamics of TEs. We performed 100 simulations for three values of insertion bias : −50, 0, and 50 ($transposition\ rate\ u=0.1$; neutral insertions). Previous work established that TE invasions typically proceed through three phases: rapid, shotgun, and inactive [23]. In the rapid phase, the TE spreads in the population unhindered by the host defense (Fig. 1C (green color)). During the shotgun phase (yellow color), there are segregating piRNA cluster insertions that are controlling the spread of the TE but they have not reached fixation in the population. In the final inactive phase, the population has fixed piRNA producing loci which are sufficient to entirely prevent transposition of the TE. When the first piRNA cluster with a TE insertion reaches fixation, this phase begins. In the initial phase of the simulations, there is no selection against transposition, so the piRNA cluster insertion reaches fixation through drift. Figure 1C illustrates the movement through these phases for three values of insertion bias. As expected, we found that an insertion bias has a marked influence on invasion dynamics, where, for example, the number of insertions per individual decreases with the insertion bias (Fig. 1C).

**Fig. 2:**
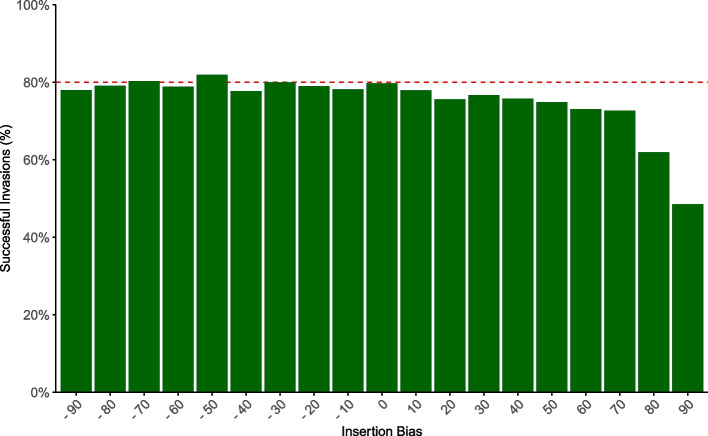
The effect of insertion bias on the probability that a TE will establish in the population. The dotted red line indicates the approximate theoretical expectation that a TE will establish in a population across all simulations. Overall, insertion bias does not significantly affect the likelihood of establishment for most values tested. A binomial test shows a significant deviation from the theoretical expectation only occurs for very high positive biases (see Additional file 1: Table S3 for *p* values)

The first critical step after horizontal transfer of a novel TE to a naive population is establishment in the new population [46]. Especially at early stages of a TE invasion, when TE copy numbers are low, a newly invading TE is likely to be lost by genetic drift. Since the probability of establishment decreases with the transposition rate ($p\approx 2u$ where *u* is the transposition rate), self-regulation of TEs by limiting their activity will reduce the rate of establishment. Here we speculate that an insertion bias into piRNA clusters may be a form of self-regulation that avoids this problem, as the TE will initially (i.e., in the absence of cluster insertions) have an uninhibited transposition rate. Only when the TE attains high copy numbers, i.e., is well established in the populations, cluster insertions will emerge that reduce the activity of the TE. We thus first tested whether the insertion bias affects the rate of establishment. The chances of establishment of a TE for invasions starting with a single segregating insertion are fairly low, which makes it hard to see further reduction due to an insertion bias. We thus started the simulations with 10 insertions to elevate the range of the observed values. We say that a TE is established if it persists for at least 500 generations. Interestingly, we found that the insertion bias has little impact on the chances of establishment, unless the bias is very high (over 60–70%, see Fig. 2). This suggests that a moderate insertion bias into piRNA clusters ($<60 \%$) does not reduce the chance of a TE getting established in a population.

**Fig. 3:**
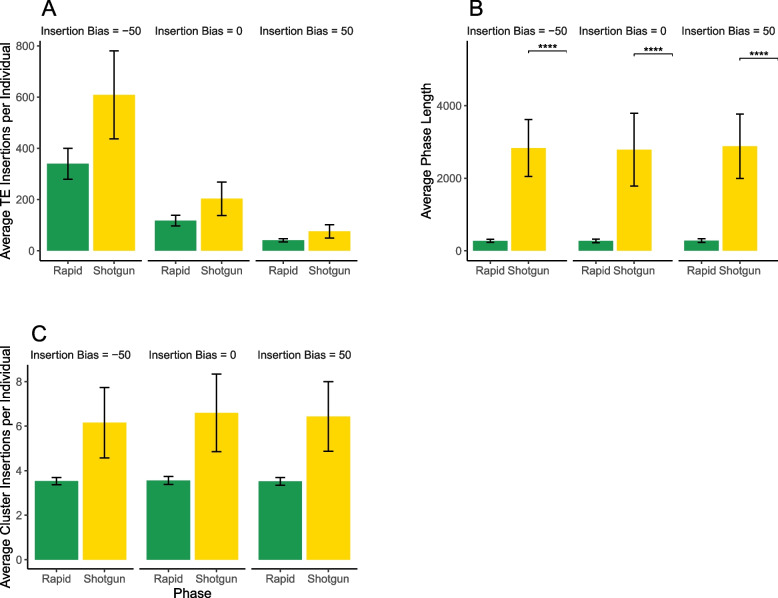
The effect of an insertion bias on TE invasion dynamics. The figure shows results for simulations with an insertion bias of −50, 0, and +50. **A** The average number of TE insertions per individual at the end of the rapid and shotgun phases. **B** The average length of the rapid and shotgun phases of a TE invasion. The length of the rapid phase is the time until the first TE insertion into a piRNA cluster within a population. The shotgun phase is the time from the first piRNA cluster insertion until TE regulation has been established in every individual of the population. Asterisks indicate a statistically significant difference between the phase lengths (Wilcoxon test, $p < 0.05$). **C** The average number of cluster insertions per individual at the end of the rapid and shotgun phases

Next, we examined the effect of insertion bias on invasion dynamics in more detail ($u=0.1$, neutral). We first noticed that the insertion bias has a substantial effect on the number of TEs accumulating during an invasion (Fig. 3A). An increasing insertion bias leads to fewer TEs accumulating during an invasion (Fig. 3A). Therefore, the degree of bias determines the number of non-cluster insertions prior to silencing of the TE. This makes intuitive sense as a TE is randomly inserting into the genome it will take more insertions to hit a piRNA cluster when there is negative bias towards piRNA clusters or no bias. This is not unexpected, since an insertion bias conceptually has a similar effect to increasing the size of piRNA clusters. Both increasing insertion bias and larger piRNA clusters increase the likelihood that a TE will jump into a piRNA cluster. Previous work revealed that the number of TE insertions accumulating during TE invasions depends largely on the size of piRNA clusters (where large clusters leads to fewer TEs accumulating during an invasion) [23]. Therefore, it is expected that an increasing insertion bias has a similar effect as larger piRNA clusters.

Next, we investigated the effect of insertion bias on the length of the TE invasion phases. We hypothesized that an insertion bias into piRNA clusters would lead to quicker suppression of TE transposition, because it should take fewer total insertions before one occurs in a cluster and silences transposition. However, this is not what we found. Figure 3B reveals that the average duration of the rapid and shotgun phases (the periods before TE inactivation) remains relatively constant across different bias levels. While there is slight variation in values and ranges, the mean phase lengths are essentially similar. This is in agreement with previous work where the length of the phases was not significantly depend on the size of piRNA clusters [23] which is conceptually similar to an insertion bias. This can be explained by the fact that TE copy numbers at early stages of an invasion increase exponentially, such that cluster insertions will rapidly emerge in all simulated scenarios.

A similar observation (Fig. 3C) can be made regarding the average number of cluster insertions per diploid individual; there is slight variance, but it does not vary significantly with bias. This is perhaps counter-intuitive, as one might expect more cluster insertions with increasing insertion bias. However, it needs to be considered that in our model TE activity stops in individuals with one (or more) cluster insertions, thus preventing further accumulation of TE copy numbers. The number of cluster insertions necessary to stop an invasion remains at about four, consistent with all previous simulations of TE invasions [6, 23]. This is true regardless of the fact that a single insertion is sufficient to silence TE transposition. Recombination among cluster insertions results in a fraction of individuals that do not carry a cluster insertion and thus the TE is able to maintain low levels of activity in the population. This will increase the average number of cluster insertions until most individuals carry about four insertions. Changing the insertion bias of the TEs did not have an effect on the average number of cluster insertions necessary to halt a TE invasion. This is again consistent with previous work where the size of piRNA clusters did not have an effect on the number of cluster insertions [23].

To summarize, in neutral simulations an insertion bias decreases the number of TEs accumulating during an invasion but has little effect on the length of the invasion phases or the number of cluster insertions when the TE is silenced.

### Insertion bias affects the fitness of the population

Under neutral conditions, insertion bias reduces the total number of insertions per individual in a population. However, TE insertions may be negatively selected, so we wanted to understand how an insertion bias might affect the impact of an invasion on organismal fitness. In particular, we speculate that an insertion bias may reduce the fitness burden that TEs pose to hosts, which could then indirectly benefit the TE.

The classic literature on TE invasions published prior to the discovery of piRNA defense was able to show that negative selection has a substantial impact on the dynamics of TE invasions, controlling TE invasion regardless of host silencing [47]. However, it has not been explored how negative selection will impact TE invasions in the presence of host silencing and insertion bias [23].

**Fig. 4:**
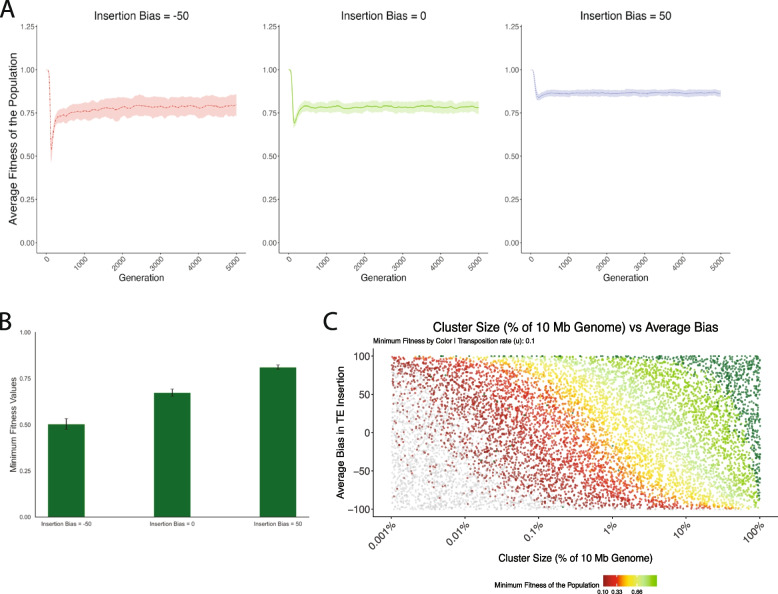
Fitness dynamics during TE invasions with varying insertion biases. **A** Average population fitness over generations for different TE insertion biases. Lines show mean fitness; shaded areas represent standard deviations. **B** Minimum population fitness ($min\_w$) achieved during invasions for three TE bias levels. **C** Population fitness mapped against piRNA cluster size (x-axis) and average TE insertion bias (y-axis). Color indicates fitness value, ranging from dark red (lowest fitness, $min\_w < 0.01$) through red ($min\_w < 0.1$), yellow ($min\_w < 0.33$), to green (highest fitness, $min\_w = 1$). Dark green points indicate populations where no TEs are left ($fail-0$); light gray points represent populations with fitness too low ($fail-w$, extinction)

To explore this question, we introduced deleterious effects of TE insertions into our simulations. We simulated a linear fitness cost of TE insertions $w = 1 -{x * n}$ where *w* is the individual fitness, *n* is the number of insertions, and *x* is the fitness cost of individual insertions. Negative selection alters the invasion dynamics of TEs considerably compared to a neutral scenario [23]. Under neutrality, TE copy numbers increase rapidly in the population early in the invasion, followed by a plateau as more piRNA cluster insertions are introduced. Depending on the extent of the negative effect (*x*), three principal outcomes are feasible. First, if negative effects are strong ($x>u$), a TE may not be able to invade as all copies are quickly purged from the population. Second, if negative effects are small ($Ne*x<1$) then the invasion will resemble a neutral scenario. Third, for intermediate values a TE will be able to spread in a population until the host defense and negative selection controls the TE (TSC-balance [23]). Figure 4A presents average fitness across generations, with standard deviations shown in lighter colors. Initially, a TE will quickly multiply in a population lowering the average fitness. As piRNA cluster insertions arise and the TE is silenced, negative selection purges the TE insertions from the population and fitness recovers. Please note that piRNA cluster insertions were still subject to negative selection in this scenario, thus fitness does not recover to 1.

In this context, we refer to minimum fitness as the lowest fitness of individuals during the TE invasion. This can also be thought of as the maximum fitness burden of the TE during an invasion. Minimum fitness is an important parameter, given the possibility that TE invasions could drive local population extinctions [48, 49]. Our observations indicate that the insertion bias indeed reduces the maximum fitness burden of TEs (Fig. 4A). This is intuitive given our previous result which found that higher bias results in a lower total number of insertions in each individual. Without selection against TE insertions, higher bias results in fewer TE insertions per individual prior to silencing of the TE. When these TEs have a negative fitness cost, it results in a higher overall fitness and thus a lower fitness burden.

Figure 4B further illustrates this maximum TE burden by showing minimum fitness during invasions of TEs with three different biases. The lowest value corresponds to a −50 bias, which which increases as the bias increases. This demonstrates that lower bias is more costly for the population. A key finding, depicted in Fig. 4C, explores population fitness in a 2D parameter space of piRNA cluster size (x-axis) and average TE insertion bias (y-axis), with fitness values color-coded. We found that for small piRNA clusters the minimum fitness can drop below 0.1. We assume that these populations cannot persist and thus will go extinct. This confirms previous observations [34] that piRNA clusters require a minimum size to control TE invasions. We note that increased insertion bias into piRNA clusters may compensate for smaller cluster sizes. Population fitness increases with cluster size and average bias, while negative bias leads to extinction even with large cluster sizes. For a successful invasion to occur, the TE’s insertion properties must be within a range (a “sweet spot”) that allows for population survival.

In summary, we found that an insertion bias reduces the fitness burden of TEs to hosts. Furthermore, a strong bias against piRNA cluster insertions could lead to extinction of populations.

### Invasion dynamics of multiple TEs with different insertion bias

Two opposing sets of selective pressures can influence TE copy number. On one hand, selection can favor TE proliferation even at a cost to host fitness. On the other hand, TEs with some form of self-regulation might result in a reduction of this fitness cost, increasing host fitness and allowing the TE to hitchhike to higher copy numbers. Given these competing outcomes, it is not intuitively clear which dynamic will lead to the most successful TE. We speculate that an insertion bias into piRNA clusters could be an effective form of self-regulation, as it allows TEs to spread rapidly when copy numbers are low, while the emergence of cluster insertions at higher copy numbers limits the damage to the host.

To test this hypothesis, we performed “pairwise-competitions” of TEs with two different insertion biases. We asked the question, under what genomic and evolutionary conditions might TEs with higher insertion bias towards piRNA clusters stabilize at higher copy number than those with lower bias? Hence, we performed simulations with two different TEs (in terms of insertion bias) that jointly invade a population.

To trigger the invasions, we introduced 100 copies of each TE at random positions. We assume that both TEs have identical properties (transposition rate $u=0.1$, negative effect) except for the bias into piRNA clusters. Furthermore, we have used a population size of $N=1000$ and 100 replicates for each scenario.

Importantly, we also assumed that insertion in a piRNA cluster silences both TEs. This is justified as we assume that insertion bias into a piRNA cluster may gradually evolve in the TE by mutations, and a few mutations may be sufficient to alter the insertion bias but they will not allow the TE to escape the host defense (e.g., piRNAs act broadly over a wide range of the TE). For example, it has been argued that up to 10% sequence divergence is tolerated between piRNAs and the silenced TE [50–52]. After 500 generations, we recorded the copy numbers of both TEs and scaled them together into a single parameter ranging between −1 and 1, for comparison.

**Fig. 5:**
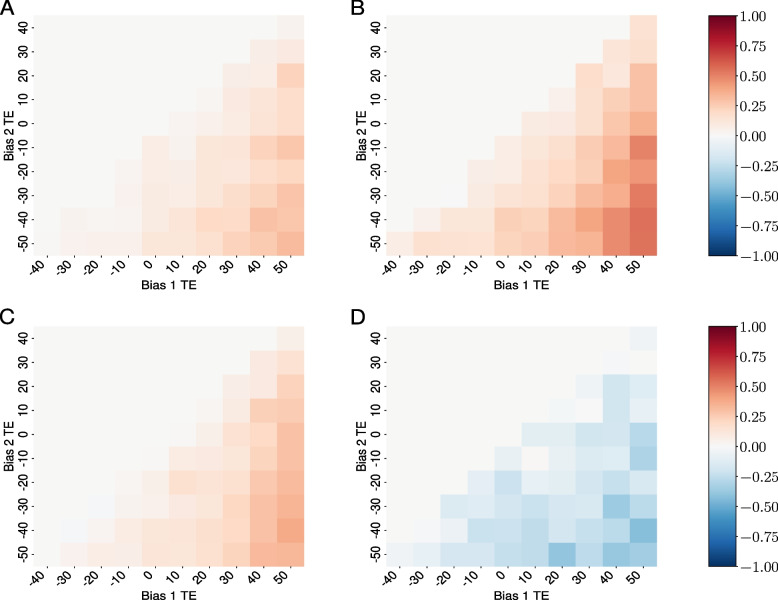
Competition dynamics between TEs with different insertion biases across varied genomic contexts. **A**–**D** correspond to scenarios detailed in Table 1. Color scale represents competitive outcomes: red indicates dominance of less biased TEs, blue shows dominance of more biased TEs, white represents equal competition, and gray dots indicate absence of both TE types. Scale calculated as $S = 2 \cdot \frac{Y}{X+Y} - 1$, where *X* and *Y* represent average total insertions for more (x-axis) and less (y-axis) biased TEs, respectively

As a control, we started with neutral simulations. In this scenario, insertion bias into a cluster is of no benefit to the TE, since TE insertions have no adverse effects on host fitness. We thus expect that TEs that avoid piRNA clusters out-compete TEs with a higher preference for clusters. We modeled two scenarios, one with recombination (random assortment among five chromosomes and crossovers occurring at a rate of 4 cM/Mbp) and one without recombination (a single non-recombining chromosome; Table 1). As expected in both scenarios, we consistently observed (Fig. 5) that TEs with lower insertion bias toward piRNA clusters obtained higher copy number than those with higher bias.

**Table 1:** Overview of competitive TE invasion simulations: Fig. 5

Scenario	Selection	Genome structure	Premise and rationale
A	Neutral	5 chr, 5 clusters	Baseline for complex genome with distributed insertion targets
B	Neutral	1 chr, 1 cluster, 0 RR	Simplified genome with concentrated target, no recombination
C	Negative	5 chr, 5 clusters	Selection in complex genomic environment
D	Negative	1 chr, 1 cluster, 0 RR	Extreme case: selection pressure, simple genome, no recombination

Note: In all scenarios: *N* = 1000, initial TE population = 100, 100 replications, 500 generations, *u* = 0.1. Negative selection coefficient *x* = 0.01. *chr* chromosomes, *RR* recombination rate

Next, we introduced negative selection against TEs ($x=0.01$) and again performed simulations in a scenario with and without recombination. If our hypothesis holds (an insertion bias is beneficial for the TE), we expect that TEs with a bias attain higher copy numbers than TEs with a lower bias. We solely observed this in the scenario without recombination. In the more biologically relevant scenario (several recombining chromosomes), we consistently observed that TEs with lower insertion bias towards piRNA clusters obtained higher copy number than those with higher bias. This shows that our hypothesis that an insertion bias is beneficial to the TE does not hold, except for the scenario without recombination. This is in agreement with previous works suggesting that self-regulation of TEs could evolve in the absence of recombination [8].

In summary, insertion bias into piRNA clusters is generally not favored in recombining genomes under purely deleterious per-copy effects. We further examined a positive-selection scenario by introducing a direct benefit parameter (*b*). When $b>0$, higher cluster insertion bias becomes advantageous (see Additional file 1: Table S4 and Additional file 1: Fig. S8.1).

## Discussion

In this manuscript, we explored the possibility that under different conditions in a population it could be beneficial to a TE to evolve an insertion bias into piRNA clusters. Our results, as illustrated in Figs. 1, 2, 3, 4, and 5, reveal a new insight about TE invasion dynamics and their impacts on host fitness.

While many TEs do not empirically appear to have an insertion bias into piRNA clusters, some TEs such as the *P*-element show a strong insertion bias. Recent studies have revealed that the relevance of insertion bias varies among different TEs and environmental conditions. The *P*-element in *Drosophila* has been shown to have a stronger insertion bias into telomere-associated sequences (TAS), which are important piRNA clusters, under hot conditions compared to cold conditions [31]. Some somatic TEs, like *gypsy* in *Drosophila*, may have an insertion bias into specific piRNA clusters such as the *flamenco* locus [23]. In certain cases, the direction of TE insertion into piRNA clusters has been found to correlate with the sense/antisense bias in piRNA production, suggesting that insertion bias can influence piRNA-mediated defense mechanisms [53].

While higher TE insertion rates into piRNA clusters have been observed in *Drosophila*, similar biases have not been consistently described in mammals, indicating potential differences in TE-host dynamics across species [54]. The important role of insertion preference in the invasion trajectory of TEs has been further emphasized by recent studies [48], building upon earlier work on the evolution of self-regulated transposition [8]. These findings, in essence, highlight the complexity and variability of TE insertion biases across different TE families, host species, and environmental conditions. The observed high insertion bias might confer unexpected benefits to both TEs and hosts. While TE insertions are generally considered costly to the host, a higher bias towards piRNA clusters could mitigate this cost by concentrating insertions in genomic regions already dedicated to TE regulation. This mechanism could allow TEs to persist in the genome while minimizing disruption to essential host genes.

These findings suggest that the observed bias of *P*-elements towards *X-TAS* may represent an evolutionary trade-off that balances TE propagation with host damage. In genomic environments where silencing is efficient and recombination is limited, targeting piRNA clusters could provide TEs with a “safe harbor” or “sweet spot” for insertion, allowing them to persist in the population while potentially contributing to the host’s defensive repertoire. The important role of insertion preference in the invasion trajectory of TEs has been further emphasized by recent studies [48], building upon earlier work on the evolution of self-regulated transposition [8]. These findings, in essence, highlight the complexity and variability of TE insertion biases across different TE families, host species, and environmental conditions. This study not only sheds light on the specific case of *P*-elements and *X-TAS* but also broadens our understanding of the evolutionary forces shaping TE-host interactions across diverse genomic landscapes. The interplay between insertion biases, environmental conditions, and host defense mechanisms reveals a complex evolutionary “arms-race” between TEs and their hosts, with implications for genome evolution and the maintenance of genomic stability.

An insertion bias into piRNA clusters is a form of self-regulation that was not previously explored [8]. An insertion bias into piRNA clusters appeared to be a plausible mechanism for self-regulation, as it avoids several problems associated with other forms. In particular self-regulation of TE activity will lower the chances of establishment in a novel population. Reduced rate of establishment will threaten the long-term persistence of a TE. We showed that an insertion bias into piRNA clusters does avoid this problem, as the effect on the establishment is minor (Fig. 1). We also demonstrated that higher insertion bias towards piRNA clusters correlates with reduced TE accumulation. The fitness dynamics presented in Fig. 4 highlight the complex relationship between TE bias, piRNA cluster size, and host fitness, with higher biases typically resulting in less fitness reduction. This supports our idea that an insertion bias may reduce harm to the host. However, contrary to expectations our competition simulations (Fig. 5) revealed that under most genomic contexts, lower-bias TEs obtain higher copy numbers than their high-bias counterparts. An insertion bias into piRNA clusters was solely beneficial (in terms of final copy numbers) in a scenario without recombination. This is in agreement with previous works showing that self-regulation of TEs might typically solely evolve in the absence of recombination [8]. Our work implies that in recombining organisms under purely deleterious per-copy effects, selection favors TEs that avoid piRNA clusters. However, we further showed that a bias against piRNA clusters will lead to elevated rates of host extinction, where the load of deleterious TEs cannot be kept in check by negative selection anymore.

This raises the question as to why more populations do not go extinct from TE invasions. There are several possible explanations. First, it is possible that an insertion into a piRNA cluster does not trigger the host defense. This hypothesis aligns with recent discoveries about the complexity and adaptability of piRNA-mediated defense systems. One study showed that even after the removal of three major piRNA clusters, TEs remained effectively silenced, suggesting a robust redundancy in the system [33]. As an alternative, it was suggested that siRNAs are mediating the conversion of TEs into piRNA producing loci. Also other forms of host defense may protect against extinctions such as KRAB-ZNFs or the hush silencing in humans. Second, it is also possible that TEs cannot evolve to avoid piRNA clusters. The high variability of piRNA clusters among species, in characteristics such as sequence composition, size, and chromatin state, may mean there are few consistent genomic or epigenomic cues that would allow a TE to reliably distinguish cluster from non-cluster DNA. Third, recent work by [29] demonstrated the crucial role of paramutation, a mechanism distinct from piRNA clusters, in the dynamics of TE silencing. In the context of TEs, paramutations refers to the conversion of a regular TE insertions into piRNA producing loci. This process is typically mediated by maternally transmitted piRNAs. The emergence of abundant piRNA producing loci due to paramutations may prevent extinctions.

These insights highlight the complex co-evolutionary dynamics between TEs and their hosts, suggesting that what appears costly or parasitic at one level might confer unexpected benefits at another. Future research could focus on experimentally testing these hypotheses, perhaps by competing TEs with (*P*-element) and without insertion bias in experimental populations of model organisms and observing the long-term effects on both TE proliferation and host fitness across varying genomic architectures.

## Conclusions

Our population genetics based forward simulations show that insertion bias into piRNA clusters alters transposable element invasion dynamics by changing the number of TE copies in individuals before silencing occurs. While insertion into piRNA clusters reduces the deleterious effects on host populations, we found that TEs avoiding piRNA clusters consistently out-compete TEs with cluster-biased insertion in recombining genomes.

Insertion bias was only beneficial to TEs under highly restrictive conditions: negative selection against TEs combined with the absence of recombination. This finding suggests that the hypothesis of evolved strategic insertion may not hold for most natural scenarios.

The empirically observed insertion biases in natural TE families, such as the *P*-element’s preference for *X-TAS*, might reflect mechanistic constraints or byproducts of other genomic targeting preferences rather than adaptive self-regulation strategies. However, we observe that strong bias against piRNA cluster insertions leads to elevated rates of host population extinction, where the burden of deleterious TEs cannot be controlled by negative selection alone.

This raises important questions about the evolutionary balance in TE-host systems. The rarity of population extinctions from TE invasions in nature suggests that other mechanisms, such as robust redundancy in piRNA defense systems, paramutation-mediated silencing, or constraints on TE evolution, may prevent the most catastrophic outcomes predicted by our models.

## Methods

### Simulation software

To simulate TE invasions with insertion bias, we developed a novel branch (“insertionbias”) for the previously developed simulation software (invadego (v0.1.3)) [29]. This software performs individual-based forward simulations of TE invasions in populations of diploid organism using discrete and non-overlapping generations. Every TE insertion has two properties, (i) a genomic position (integer) in the half-open interval [0, *g*), where *g* is the genome size, and (ii) and an insertion bias (byte) into piRNA clusters. Note that it is thus possible to simulate TEs with different insertion biases in the same genome. The TE insertions in a haploid genome are represented as a dictionary where the position acts as key and the bias as value. Thus, a diploid individual carries two separate dictionaries of TE insertions. Each chromosome occupies a unique non-overlapping territory in the genomic interval [0, *g*), where every TE insertion is part of exactly one chromosome. piRNA clusters occupy sub-regions of each chromosome. TE insertions may be a part of none or one piRNA clusters. We opted to model the insertion bias as a discrete integer value from −100 to +100 (represented as a single byte, to minimize memory consumption), where 0 is unbiased, −100 is a strong bias against piRNA clusters (no insertions in piRNA clusters), and +100 is a strong insertion bias into piRNA clusters (all insertions are in piRNA clusters). The probability of a novel TE inserting into a piRNA cluster ($p_{c}$) can be computed from the bias (*b*) and the genomic proportion of piRNA clusters (*f*). For example, if piRNA clusters account for 3.5% of the genome, as in *Drosophila*, then $f=0.035$.

$$\begin{aligned} a & = (b/100+1)/2 \\ s & = a*f + (1-a)*(1-f) \\ p_{c} & = a*f / s \end{aligned}$$

The resulting probability ($p_c$) will be a value between 0 and 1. Note that in the absence of an insertion bias ($b=0$) the probability to insert into a piRNA cluster is identical to the genomic fraction of the piRNA cluster ($p_{c}=f$).

Each individual has a fitness *w*, which solely depends on the number of TE insertions $w=1-xn$, where *x* is the negative effect of a single TE insertion and *n* is the number of TE insertions per diploid individual. Simulations with neutral TE insertions can be performed using $x=0$. The fitness determines the mating probability (i.e., fecundity selection). We simulated hermaphrodites that may randomly mate with other hermaphrodites. Each parent generates a single gamete that is passed to the offspring. To create a gamete, first recombination and random assortment among chromosomes are simulated and then novel transposition events are introduced into the recombined gamete. We assumed that TEs multiply with a given transposition rate *u*, which is the probability that a TE insertion will generate a novel insertion in the next generation. A transposition rate of zero ($u=0$) was used for individuals carrying an insertion in a piRNA cluster. To avoid excessive computation times, we calculated the number of novel insertion sites for each gamete based on a Poisson distributed random variable with $\lambda =u*n/2$. Based on the probability of jumping into a piRNA cluster ($p_c$ see above), we randomly distributed novel insertions either within or outside of piRNA clusters. If a site was already occupied, the novel insertion was ignored.

Our software allows the user to provide a wide range of different parameters such as the number of chromosomes, the size of the chromosomes, the size of the piRNA clusters, the recombination rate, the transposition rate, the population size, the number of generations, the number of TE insertions in the base population, the negative effect of TEs, and a flag indicating whether or not cluster insertions are selectively neutral. For the base population, it is possible to provide a file with the position and the bias of the TE insertions.

The novel tool was thoroughly tested with unit-tests. We further validated the correct implementation of our software to confirm that it correctly models population forces such as recombination, drift, and selection (Additional file 1: Figs. S1–S7). For example, theoretically a proportion of TE insertions should reach fixation due to genetic drift depending upon the population size. These expectations were met and all of the simulations performed to validate the model are described in the supplement. Additionally, we verified that the software accurately models insertion bias as specified, illustrated in Fig. 1B. The simulations, analysis, and figures for visualization from this work have been documented and deposited on GitHub. Ninety-two to 100% of the invasions were stopped after 5000 generations and all after 10,000 generations (Additional file 1: Table S1; Fig. 1C)

### Simulations and data analysis

For simulations, we have used several default conditions—five chromosome arms of 10 Mbp each, a recombination rate of 4 cM/Mbp, piRNA clusters of 300 kb (3% of the genome), a population size of 1000, transposition rate of 0.1, and a base population with 100 randomly inserted TEs. The last parameter is to avoid losing TEs to genetic drift [23, 29, 46]. For every simulation, we performed 100 replicates. We initially simulated TE insertions with no negative selection, but later incorporated scenarios with selection against TE insertions.

The output of all of the simulations was visualized in R using ggplot2 [55], Seaborn [56], and matplotlib [57]. Simulations output a large amount of data; therefore, we also used DuckDB [58] for data management.

## Supplementary information

Additional file 1. Supplementary Information. PDF file containing validation analyses, statistical tests, and additional simulation results. Sections S1-S8: S1: Invasion dynamics validation; S2: Genetic drift validation; S3: piRNA cluster size analysis; S4: Recombination and linkage disequilibrium decay; S5: Insertion bias implementation verification; S6: Selection scenarios; S7: Additional validation tests; S8: Direct cluster benefit simulations. Tables S1-S4: S1: Invasion stopping statistics; S2: Establishment probabilities; S3: Statistical test *p*-values; S4: Direct benefit competition outcomes. Figures S1.1-S8.1: S1.1-S1.2: Invasion dynamics; S2.1: Drift effects; S3.1-S3.2: Cluster size impact; S4.1-S4.2: Recombination effects; S5.1-S5.6: Bias implementation; S6.1-S6.11: Selection scenarios; S7.1-S7.6: Additional validations; S8.1: Direct benefit results.

## Data Availability

Invadego insertion module is available at GitHub: https://github.com/RobertKofler/invadego/tree/insertionbias. The population genetics validations are documented at https://github.com/shashankpritam/Insertion-Bias-TE.

## Abbreviations

TE: Transposable element
TEs: Transposable elements
piRNA: PIWI-interacting RNA
piRNAs: PIWI-interacting RNAs
kb: Kilobase
Mbp: Megabase pair
cM: Centimorgan
bp: Base pair
