# Supplementary material for: The impact of insertion bias into piRNA clusters on the invasion of transposable elements
Source: BMC Biol. 2025 Aug 15;23:258. doi: 10.1186/s12915-025-02370-0 (PMC12357481; doi:10.1186/s12915-025-02370-0)
Supplement: Supplementary file 1 — Additional file 1. Supplementary Information. PDF file containing validation analyses, statistical tests, and additional simulation results. Sections S1-S8: S1: Invasion dynamics validation; S2: Genetic drift validation; S3: piRNA cluster size analysis; S4: Recombination and linkage disequilibrium decay; S5: Insertion bias implementation verification; S6: Selection scenarios; S7: Additional validation tests; S8: Direct cluster benefit simulations. Tables S1-S4: S1: Invasion stopping statistics; S2: Establishment probabilities; S3: Statistical test p-values; S4: Direct benefit competition outcomes. Figures S1.1-S8.1: S1.1-S1.2: Invasion dynamics; S2.1: Drift effects; S3.1-S3.2: Cluster size impact; S4.1-S4.2: Recombination effects; S5.1-S5.6: Bias implementation; S6.1-S6.11: Selection scenarios; S7.1-S7.6: Additional validations; S8.1: Direct benefit results. [file 12915_2025_2370_MOESM1_ESM.pdf]

Supplementary Data

Shashank Pritam

July 29, 2025

Contents

I Supplementary Figures 6

S1 Invasion 6

S2 Drift 8

S3 piRNA clusters 8

S4 Recombination 10

S5 Insertion Bias 11

S6 Selection 15

S7 Insertion Validation 23

S8 TE Competition with Direct Fitness Benefit 28

II Supplementary Tables 29

List of Figures

S1.1 Validation of invasion dynamics: In this simulation, we validated that the transposable element (TE) insertion in the entire population followed the expected exponential growth based on theoretical and mathematical predictions. While most invasion replicates adhered to the expected trajectory (represented by the thick black line), some replicates also show variation due to stochastic effects. 7

S1.2 Validation of invasion dynamics: Similar to the previous simulation, we validated that the transposable element (TE) insertion in the individual organism followed the expected exponential growth based on theoretical and mathematical predictions. While most invasion replicates adhered to the expected trajectory (represented by the thick black line), some replicates also exhibited variation due to stochastic effects. 7

S2.1 Validation of genetic drift: Distribution of fixed TEs in the population. The x-axis shows the number of fixed TEs, and the y-axis shows the count of replicates. 8

S3.1 Validation of piRNA cluster: TE copies in the entire population with respect to generation for different piRNA cluster sizes . . . 9

S3.2 Validation of piRNA cluster: TE copies per diploid individual with respect to generation for different piRNA cluster sizes. . . . 9

S4.1 Validation of recombination dynamics: Expected LD decay for different recombination rates. 10

S4.2 Validation of recombination dynamics: Observed LD decay for different recombination rates.. 11

S5.1 Analysis of Insertion Bias: Percentage of successful TE invasions as a function of insertion bias. The x-axis represents the insertion bias ranging from -90 to 90, while the y-axis shows the percentage of successful TE invasions. 12

S5.2 Analysis of Insertion Bias: TE insertions per diploid individual over generations for different insertion bias values. Each panel represents a specific insertion bias ranging from -90 to 90. 12

S5.3 Analysis of Insertion Bias: TE insertions within piRNA clusters at different phases for various insertion bias values. The bar plot shows the number of TE insertions in piRNA clusters before the shotgun (rapid) and before the inactive (shotgun) phases. Each panel represents a specific insertion bias value ranging from -90 to 90. 13

S5.4 Analysis of Insertion Bias: TE insertions within piRNA clusters at different phases for various insertion bias values. The bar plot shows the number of TE insertions in piRNA clusters before the shotgun (rapid) and before the inactive (shotgun) phases. Each panel represents a specific insertion bias value ranging from -90 to 90. 13

S5.5 Analysis of Insertion Bias: Box plot showing the number of TE insertions per individual for different insertion bias values. The x-axis represents the insertion bias, ranging from 0 to 90 and then from -10 to -90, with a step size of 10. The y-axis represents the number of TE insertions per individual. 14
S5.6 Analysis of Insertion Bias: Box plot showing the number of TE insertions within piRNA clusters for different insertion bias values. The x-axis represents the insertion bias, ranging from 0 to 90 and then from -10 to -90, with a step size of 10. The y-axis represents the number of TE insertions within piRNA clusters. . 15
S6.1 Validation of selection effects: Position of cluster (clu) vs non-Cluster (noclu) insertion at generation 0, 25 and 50 - selection through generations using a counter of per 10kb bin in the genome. 16
S6.2 Validation of selection effects: Relative frequency of TEs in clusters (clu) and non-clusters (noclu) at generations 0 and 50 for different selection scenarios. Left: Selection on all TEs. Right: Selection only on non-cluster TEs. 16
S6.3 Validation of selection effects: Distribution of TEs in clusters (red) and non-clusters (blue) across the genome at generations 0, 25, and 50 for a single replication. The y-axis represents the count of TEs per 10kb bin, while the x-axis shows the genomic position from 0 to 1Mb. The piRNA clusters are defined in the initial part of the genome (left side). 17
S6.4 Validation of selection effects: Relative frequency of TEs in clusters (clu) and non-clusters (noclu) at generations 0 and 100 for different selection scenarios. Left: Selection on all TEs. Right: Selection only on non-cluster TEs. 18
S6.5 Validation of selection effects: Progression of TE population (y-axis) through generations (x-axis) for different selection coefficients (x) on the same population. Top left: x = 0, top middle: x = 0.1, top left: x = 0.01, bottom right: x = 0.001, bottom left: x = 0.0001. 18
S6.6 Validation of selection effects: Progression of TE population frequency through generations for x = 0. The blue line represents the average TE frequency. 19
S6.7 Validation of selection effects: Progression of TE population frequency through generations for x = 0.1. The blue line represents the average TE frequency. 20
S6.8 Validation of selection effects: Progression of TE population frequency through generations for x = 0.01. The blue line represents the average TE frequency. 20
S6.9 Validation of selection effects: Progression of TE population frequency through generations for x = 0.001. The blue line represents the average TE frequency. 21
S6.10 Validation of selection effects: Progression of TE population frequency through generations for x = 0.0001. The blue line represents the average TE frequency. 21

S6.11 Validation of selection effects: Progression of TE population frequency through generations for different selection coefficients (x) on the same population, with average TE frequency represented by blue lines. Top left: x = 0, top right: x = 0.1, middle left: x = 0.01, middle right: x = 0.001, bottom: x = 0.0001. 22
S7.1 Validation of insertion: Average cluster insertion as a function of insertion bias. The x-axis represents the insertion bias ranging from -100 to 100 with a step size of 10. The y-axis shows the average cluster insertion. The dots in the plot represent the observed values, while the orange line represents the expected values. The observed and expected values match, showing the steep exponential curve. 23
S7.2 Validation of insertion: Variability in TE insertions across different levels of insertion bias, based on data from 100 replications. The y-axis represents the relative difference between 'avcli' and 'pc' columns, expressed as a percentage. The x-axis represents the categorical 'Insertion Bias' variable. Each boxplot summarizes the distribution of 'TE_insertions' for each level of 'Insertion Bias', indicating the median, interquartile range, and potential outliers. 24
S7.3 Validation of insertion: Mean of average cluster insertion across various insertion bias levels, with error bars indicating standard deviation. The orange line represents the expected values. 25
S7.4 Validation of insertion: The same data as Figure S7.3, but with a logarithmic transformation applied to the y-values. The log scale helps to better visualize the variability in the data (shown by the error bars) and the deviation of the observed values (blue points) from the expected values (orange line). 26
S7.5 Validation of insertion: Average TE insertions (y-axis) and insertion bias (x-axis) for a single replicate. The x-axis represents the insertion bias varying from -100 to 100, while the y-axis depicts the average TE insertions for each bias level. Each data point represents the average TE insertions for a specific bias level for replicate 1. 27
S7.6 Validation of insertion: All replicates' mean average TE insertions (y-axis) against the insertion bias (x-axis). The plot includes error bars representing the standard deviation for the average TE insertions at each bias level. The x-axis represents the insertion bias, ranging from -100 to 100, while the y-axis depicts the mean of the average TE insertions for each bias level. 28
S8.1 The impact of a direct fitness benefit for cluster insertion on TE competition. The four scenarios (A-D) correspond to the parameters detailed in Table 4, exploring how competition shifts as the negative selection pressure (x) and cluster insertion benefit (b) are varied. The color scale represents the competitive outcome: red indicates the dominance of TEs with a lower insertion bias (more selfish), while blue indicates the dominance of TEs with a higher insertion bias (more self-regulating). The scale is calculated same as in Figure 5 in the manuscript. 29

List of Tables

1 Across the 21 insertion bias values (sampleid 0 to 20), the mean of cluster insertion is calculated for all 100 replications. The standard deviation is also calculated. We have another column named pc which is the theoretical value of average cluster insertion for a given insertion bias with a 3% cluster size. 30

2 Probability (p) that a TE jumps into a piRNA cluster depending on the insertion bias of the TE. The piRNA clusters accounts for 3% of the genome. Additionally, we show the number of expected insertions and observed with our software invadego for 1000 new TE insertions. Observed values are averaged over 1000 replicates. This demonstrates that our software correctly implements the insertion bias. 31

3 Supplementary Table 3: Statistical validation of TE establishment probability. For each insertion bias, the observed number of successful establishments out of 1000 simulation replicates was compared against a theoretical expectation of 81% using a two-sided binomial test. The 81% expectation is derived from the biological model of piRNA cluster maturation for a TE with no insertion bias, requiring it to survive approximately three generations to become established (0.9 * 0.9 = 0.81). This value serves as a baseline to test for deviations caused by insertion bias. The resulting p-values were adjusted for multiple comparisons using the Bonferroni correction. Significant deviation from the theoretical expectation is observed primarily at high positive insertion biases ($\geq +20$). 32

4 Overview of TE Invasion Simulations with Cluster Insertion Benefit 33

Part I
Supplementary Figures

This section presents the supplementary figures related to the validation steps described in the manuscript.

S1 Invasion

The purpose of this validation is to show that the simulator generates, on average, the number of insertions predicted by the following equation:

$$c_t = c_0(1+\mu)^t \tag{1}$$

where:

- c_t: number of TE copies at generation t
- c_0: number of TE copies at generation 0
- μ: transposition rate
- t: generation number

The initial conditions for the simulation are:

$$c_0 = 10$$
$$\mu = 0.1$$
$$t = 100$$

The simulation is performed on a chromosome of size 1 Mb without any piRNA clusters. A total of 500 replicates are used.

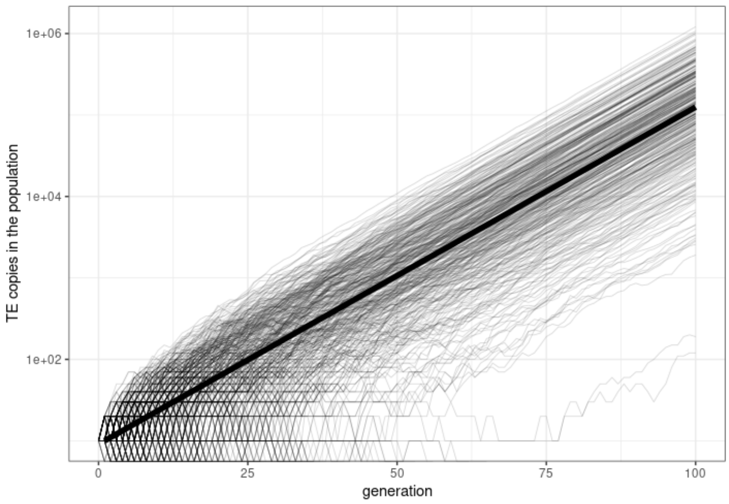

Figure S1.1: Validation of invasion dynamics: In this simulation, we validated that the transposable element (TE) insertion in the entire population followed the expected exponential growth based on theoretical and mathematical predictions. While most invasion replicates adhered to the expected trajectory (represented by the thick black line), some replicates also show variation due to stochastic effects.

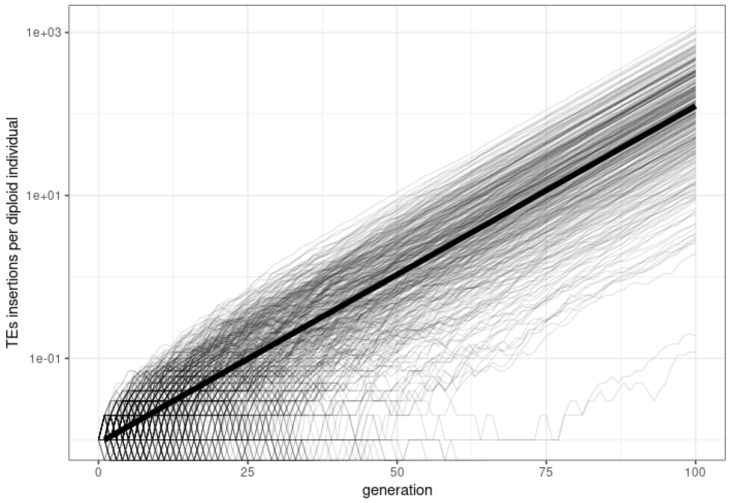

Figure S1.2: Validation of invasion dynamics: Similar to the previous simulation, we validated that the transposable element (TE) insertion in the individual organism followed the expected exponential growth based on theoretical and mathematical predictions. While most invasion replicates adhered to the expected trajectory (represented by the thick black line), some replicates also exhibited variation due to stochastic effects.

S2 Drift

To test if genetic drift is simulated correctly, we exploit a fundamental population genetics principle: the probability of fixation of a neutral singleton in a diploid organism is given by:

$$P(\text{fix}) = \frac{1}{2N_e} \quad (2)$$

where N_e is the effective population size. In the simulation results, the x-axis represents the number of fixed transposable elements (TEs) within the population, while the y-axis shows the count of replicates. We expect the distribution of fixed TEs to follow the probability of fixation given by Equation (1).

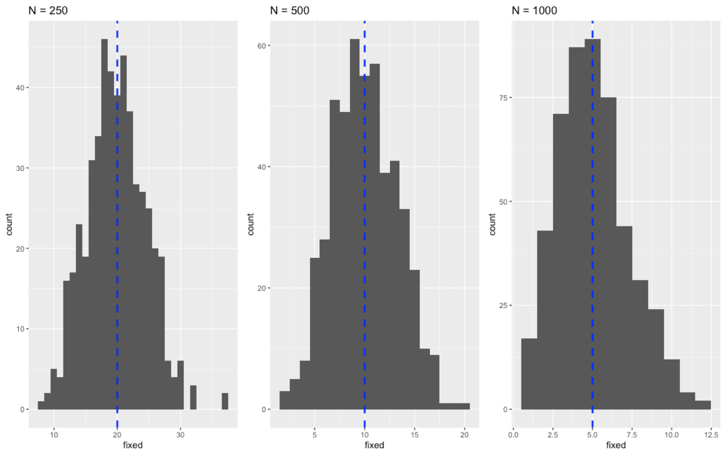

Figure S2.1: Validation of genetic drift: Distribution of fixed TEs in the population. The x-axis shows the number of fixed TEs, and the y-axis shows the count of replicates.

As shown in Figure S2.1, the observed distribution of fixed TEs in the simulated population aligns with the expected probability of fixation, confirming that genetic drift is accurately simulated in the model.

S3 piRNA clusters

In this validation, we consider three different scenarios based on the proportion of the genome composed of piRNA clusters:

- pc0: no piRNA clusters
- pc50: 50% of the genome is composed of piRNA clusters
- pc100: 100% of the genome is composed of piRNA clusters

The following starting conditions are used for all simulations:

$$c_0 = 10$$
$$\mu = 0.1$$
$$t = 100$$

The simulations are performed on a chromosome of size 1 Mb with variable piRNA cluster sizes.

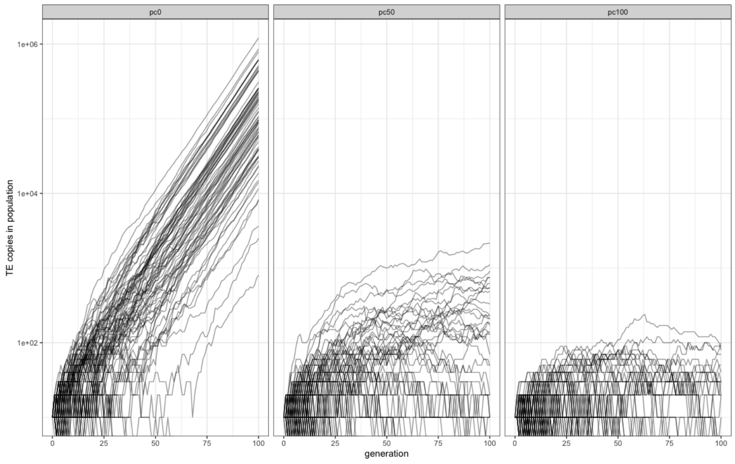

Figure S3.1: Validation of piRNA cluster: TE copies in the entire population with respect to generation for different piRNA cluster sizes

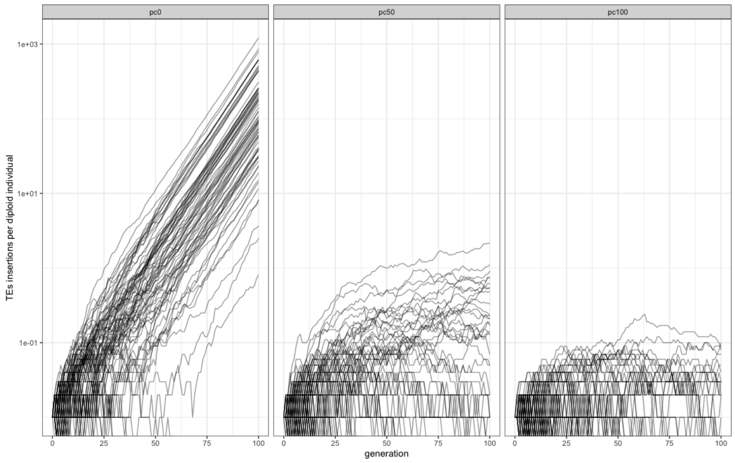

Figure S3.2: Validation of piRNA cluster: TE copies per diploid individual with respect to generation for different piRNA cluster sizes.

Figures S3.1 and S3.2 show the effect of piRNA clusters on the spread of transposable elements (TEs) in the population. Figure S3.1 depicts the total

number of TE copies in the entire population, while Figure S3.2 shows the average number of TE copies per diploid individual. Both figures demonstrate that the introduction of piRNA clusters effectively suppresses the proliferation of TEs over generations. As the proportion of the genome composed of piRNA clusters increases (from pc0 to pc100), the spread of TEs is increasingly inhibited, showing the important role of piRNA clusters in regulating TE activity.

S4 Recombination

This validation aims to test the correct implementation of recombination using linkage disequilibrium decay. Linkage disequilibrium (LD) is the non-random association of alleles at different loci in a population. The decay of LD is influenced by factors such as inbreeding and recombination rate. The number of generations needed to reach $D = 0$ is described by the equation:

$$D_n = (1 - c)^n D_0 \tag{3}$$

where n is the number of generations, D_n is the LD value at generation n, D_0 is the initial LD value, and c is the recombination rate.

All validations use a population of $N = 10000$ and an initial TE distribution of 1000, simulated for 150 generations.

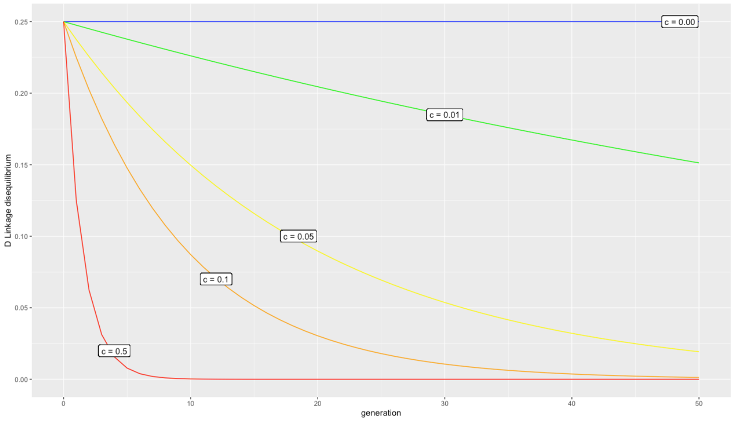

Figure S4.1: Validation of recombination dynamics: Expected LD decay for different recombination rates.

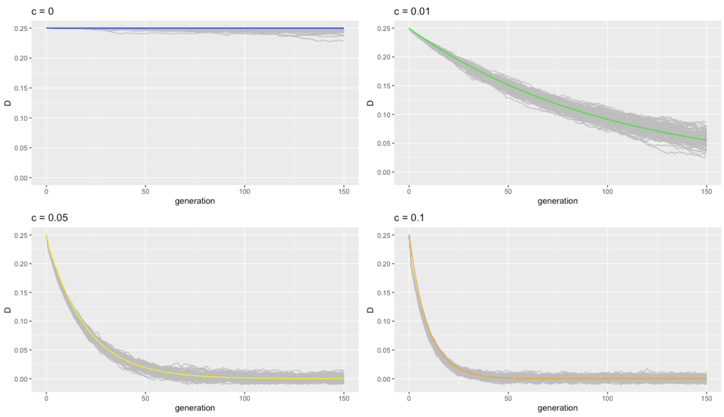

Figure S4.2: Validation of recombination dynamics: Observed LD decay for different recombination rates..

- Validation Part 1: Recombination rate = 0.00
- Validation Part 2: Recombination rate = 0.01
- Validation Part 3: Recombination rate = 0.05
- Validation Part 4: Recombination rate = 0.1

The observed LD decay (Figure S4.2) closely matches the expected decay (Figure S4.1) for each recombination rate, confirming the correct implementation of recombination in the simulation.

S5 Insertion Bias

This simulation aims to understand the effect of insertion bias on the invasion dynamics of transposable elements (TEs).

The simulation uses the following initial conditions:

- Population size: 1000
- Number of chromosomes: 5
- Chromosome size: 10 Mb
- Number of piRNA clusters: 5
- piRNA cluster size: 300 Kb
- Initial number of TEs in the population: 10

For the establishment probability simulation (Part A), 1000 replicates were used.

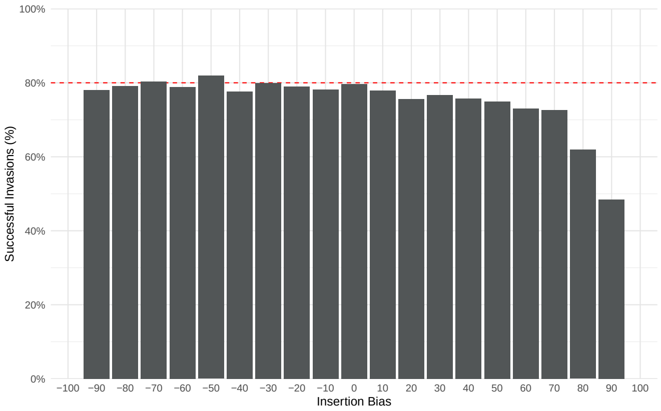

Figure S5.1: Analysis of Insertion Bias: Percentage of successful TE invasions as a function of insertion bias. The x-axis represents the insertion bias ranging from -90 to 90, while the y-axis shows the percentage of successful TE invasions.

Figure S5.1 shows the relationship between insertion bias and the percentage of successful TE invasions. The x-axis represents the insertion bias, ranging from -90 to 90, while the y-axis shows the percentage of successful TE invasions.

The main observation from this simulation is that the probability of successful TE invasion remains relatively constant (around 80%) unless the insertion bias is extremely high (over 60-70).

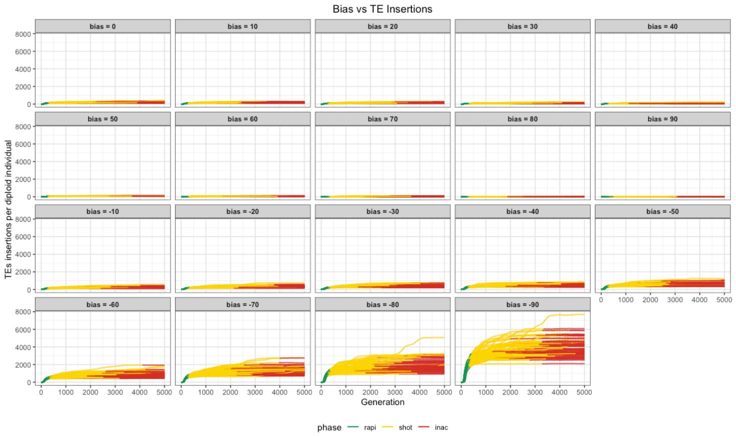

Figure S5.2: Analysis of Insertion Bias: TE insertions per diploid individual over generations for different insertion bias values. Each panel represents a specific insertion bias ranging from -90 to 90.

Figure S5.2 shows the number of TE insertions per diploid individual over generations for different insertion bias values, ranging from -90 to 90. Each panel in the figure represents a specific insertion bias value. The main observation from this simulation is that as the insertion bias moves from -90 to +90, the number of TE copies per individual decreases. This suggests that positive insertion bias

(towards piRNA clusters) leads to a reduction in the overall TE load within the population.

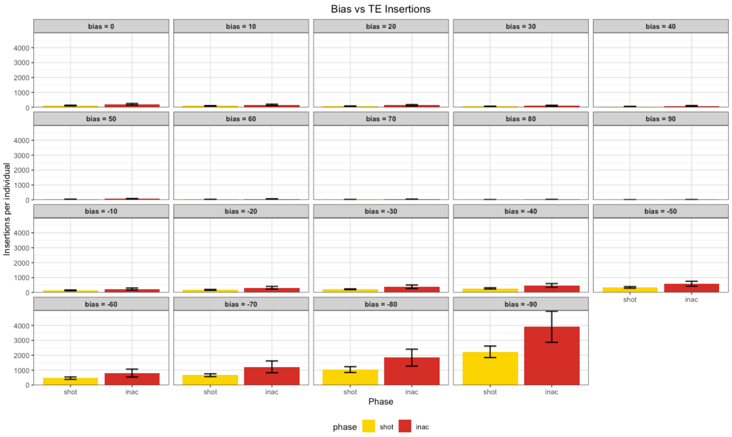

Figure S5.3: Analysis of Insertion Bias: TE insertions within piRNA clusters at different phases for various insertion bias values. The bar plot shows the number of TE insertions in piRNA clusters before the shotgun (rapid) and before the inactive (shotgun) phases. Each panel represents a specific insertion bias value ranging from -90 to 90.

Figure S5.3 presents a bar plot displaying the number of TE insertions per individual at two distinct phases: before the shotgun phase (rapid) and before the inactive phase (shotgun). The plot is divided into panels, each representing a specific insertion bias value ranging from -90 to 90.

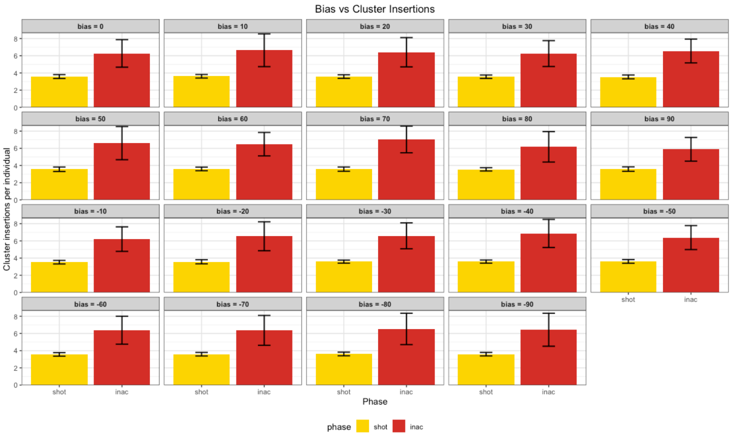

Figure S5.4: Analysis of Insertion Bias: TE insertions within piRNA clusters at different phases for various insertion bias values. The bar plot shows the number of TE insertions in piRNA clusters before the shotgun (rapid) and before the inactive (shotgun) phases. Each panel represents a specific insertion bias value ranging from -90 to 90.

Figure S5.4 presents a bar plot displaying the number of TE insertions within piRNA clusters at two distinct phases: before the shotgun phase (rapid) and before the inactive phase (shotgun). The plot is divided into panels, each representing a specific insertion bias value ranging from -90 to 90. We observe that the number of TE insertions within piRNA clusters remains consistent across different insertion bias values within each panel.

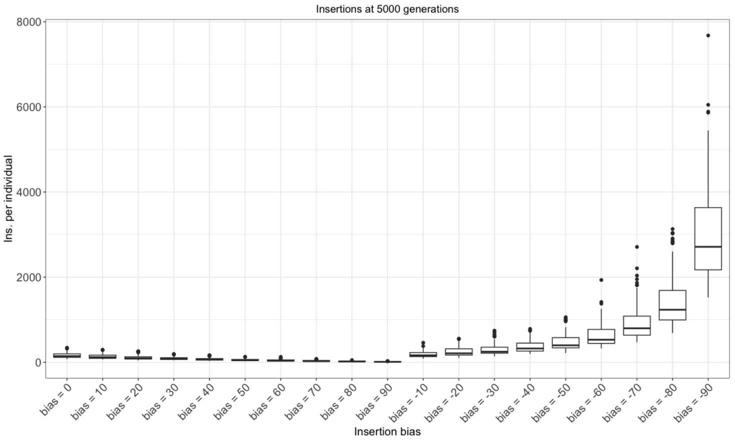

Figure S5.5: Analysis of Insertion Bias: Box plot showing the number of TE insertions per individual for different insertion bias values. The x-axis represents the insertion bias, ranging from 0 to 90 and then from -10 to -90, with a step size of 10. The y-axis represents the number of TE insertions per individual.

Figure S5.5 presents a box plot illustrating the relationship between insertion bias and the number of TE insertions per individual. The x-axis represents the insertion bias, ranging from 0 to 90 and then from -10 to -90, with a step size of 10. The y-axis represents the number of TE insertions per individual.

Starting from an insertion bias of 0, the median number of TE insertions per individual gradually decreases as the bias increases towards 90. And as the insertion bias becomes negative, starting from -10, the median number of TE insertions per individual begins to rise again. This trend continues until the insertion bias reaches -90.

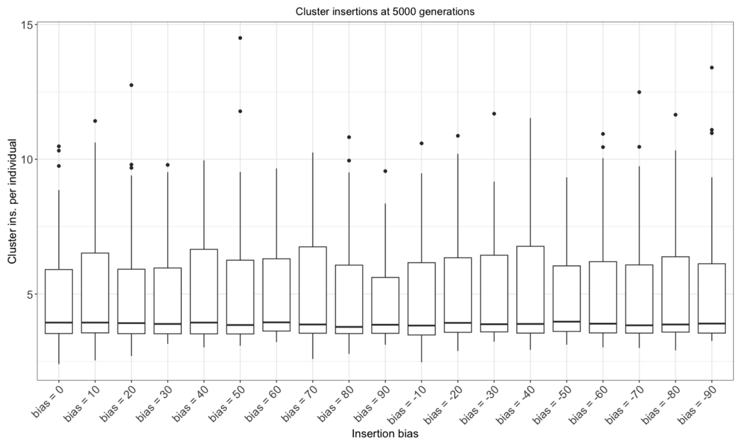

Figure S5.6: Analysis of Insertion Bias: Box plot showing the number of TE insertions within piRNA clusters for different insertion bias values. The x-axis represents the insertion bias, ranging from 0 to 90 and then from -10 to -90, with a step size of 10. The y-axis represents the number of TE insertions within piRNA clusters.

Figure S5.6 presents a box plot illustrating the relationship between insertion bias and the number of TE insertions within piRNA clusters. The x-axis represents the insertion bias, ranging from 0 to 90 and then from -10 to -90, with a step size of 10. The y-axis represents the number of TE insertions within piRNA clusters.

The box shows trend in the number of TE insertions within piRNA clusters as the insertion bias varies. Starting from an insertion bias of 0, the median number of TE insertions within piRNA clusters remains steady as the value of bias changes.

S6 Selection

In this validation, we aimed to test if selection was correctly implemented in the simulation. To do so, we tested different scenarios: selection on all TEs versus selection on non-cluster TEs.

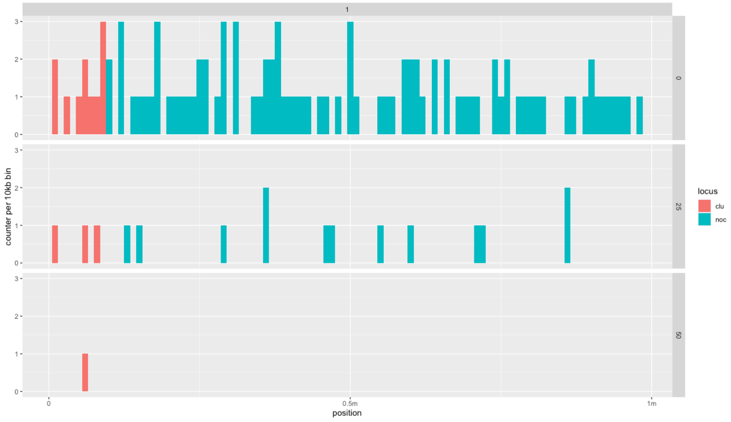

Figure S6.1: Validation of selection effects: Position of cluster (clu) vs non-Cluster (noclu) insertion at generation 0, 25 and 50 - selection through generations using a counter of per 10kb bin in the genome.

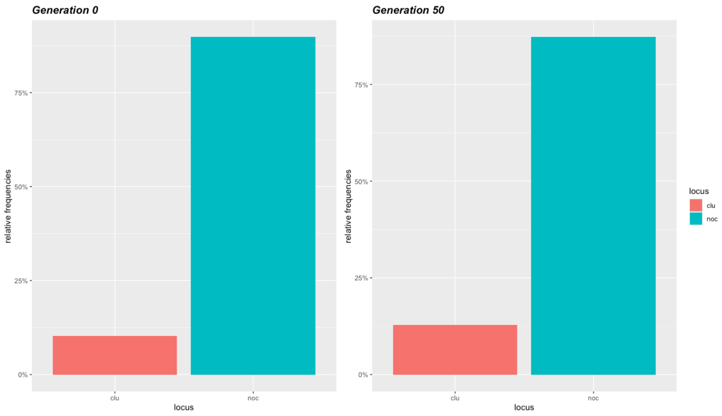

Figure S6.2: Validation of selection effects: Relative frequency of TEs in clusters (clu) and non-clusters (noclu) at generations 0 and 50 for different selection scenarios. Left: Selection on all TEs. Right: Selection only on non-cluster TEs.

Figure S6.2 shows two side-by-side bar plots comparing the relative frequency of TEs in clusters (clu) and non-clusters (noclu) at generations 0 and 50 for different selection scenarios. The plot on the left shows the results when selection is applied to all TEs, while the plot on the right displays the outcome when selection is only applied to non-cluster TEs. In both scenarios, the relative frequencies of TEs in clusters and non-clusters increase slightly from generation 0 to generation 50. Also, the overall ratio between the frequencies of TEs in clusters and non-clusters remains relatively constant across generations in both selection scenarios.

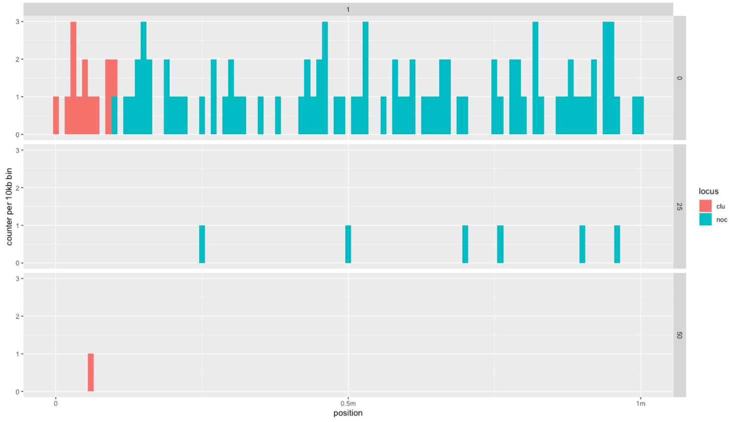

Figure S6.3: Validation of selection effects: Distribution of TEs in clusters (red) and non-clusters (blue) across the genome at generations 0, 25, and 50 for a single replication. The y-axis represents the count of TEs per 10kb bin, while the x-axis shows the genomic position from 0 to 1Mb. The piRNA clusters are defined in the initial part of the genome (left side).

Figure S6.3 show three stacked bar plots, each representing a different generation (0, 25, and 50) for a single replication of the simulation. The y-axis indicates the count of TEs per 10kb bin, while the x-axis represents the genomic position from 0 to 1Mb. The red bars denote TEs within piRNA clusters, and the blue bars represent TEs in non-cluster regions.

At generation 0, we observe a high abundance of both red and blue bars, indicating a substantial number of TEs in both cluster and non-cluster regions. The blue bars (non-cluster TEs) appear to be more numerous compared to the red bars (cluster TEs).

As we progress to generation 25, there is a noticeable change in the distribution of TEs. The blue bars are predominantly located on the right side of the plot, suggesting a shift in the distribution of non-cluster TEs towards the latter part of the genome. The red bars, representing cluster TEs, are no longer visible.

Finally, at generation 50, we observe a single red bar on the left side of the plot, corresponding to the initial part of the genome where piRNA clusters are defined in the simulation. This suggests that the cluster TEs have been largely eliminated, with only a small remnant remaining in the designated cluster region. The initial abundance of both cluster and non-cluster TEs at generation 0, followed by the shift in non-cluster TEs towards the right side of the genome at generation 25, and the eventual elimination of most cluster TEs by generation 50, shows the complex interplay between TE activity, piRNA-mediated silencing, and selection pressures.

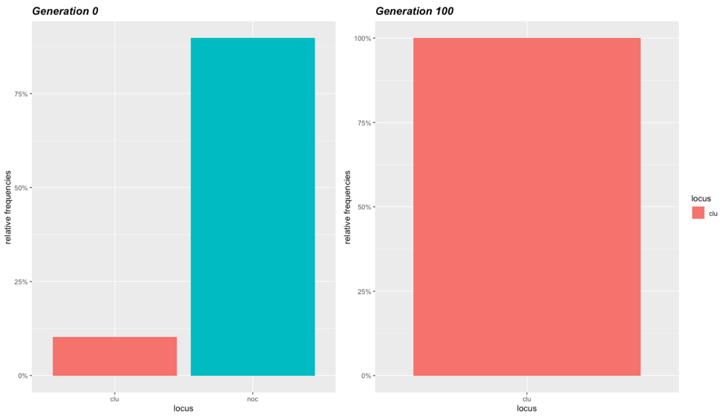

Figure S6.4: Validation of selection effects: Relative frequency of TEs in clusters (clu) and non-clusters (noclu) at generations 0 and 100 for different selection scenarios. Left: Selection on all TEs. Right: Selection only on non-cluster TEs.

Figure S6.4 presents two side-by-side bar plots comparing the relative frequency of TEs in clusters (clu) and non-clusters (noclu) at generations 0 and 100 for different selection scenarios. The plot on the left shows the results when selection is applied to all TEs, while the plot on the right displays the outcome when selection is only applied to non-cluster TEs. At generation 0, both plots exhibit a similar distribution of TEs in clusters and non-clusters, with a considerable presence of both red bars (cluster TEs) and blue bars (non-cluster TEs). This indicates that at the initial stage, TEs are distributed evenly across both cluster and non-cluster regions of the genome. However, at generation 100, a difference emerges between the two selection scenarios. In the plot, the red bars representing cluster TEs dominate the distribution, while the blue bars representing non-cluster TEs have diminished to zero.

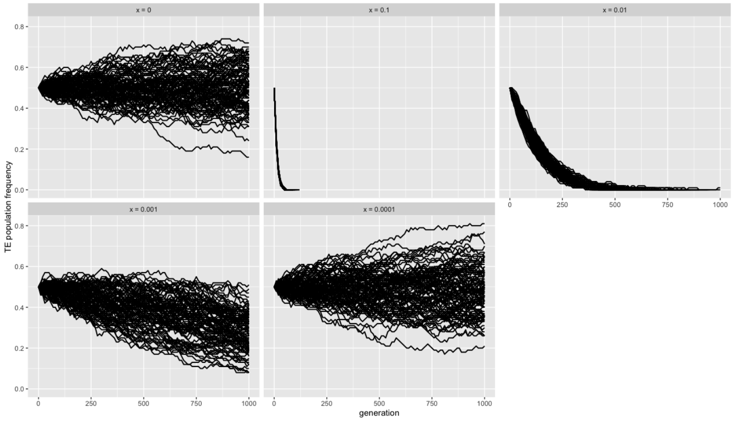

Figure S6.5: Validation of selection effects: Progression of TE population (y-axis) through generations (x-axis) for different selection coefficients (x) on the same population. Top left: x = 0, top middle: x = 0.1, top left: x = 0.01, bottom right: x = 0.001, bottom left: x = 0.0001.

Figure S6.5 presents a facet wrap plot showing the progression of TE population frequency (y-axis) through generations (x-axis) for different selection coefficients (x) on the same population. In the top left panel (x = 0), the base population is defined as 4000 TEs in the genome, and the replicates show both upward and downward trends, indicating that the TE population frequency is not biased. Moving towards the right, in the top middle panel (x = 0.1), all replicates show a decrease in TE population frequency towards 0. In the top left panel (x = 0.01), more replicates survive, but the TE count still decreases. The bottom right panel (x = 0.001) shows a similar trend, with more surviving replicates but a consistent decrease in TE frequency. The bottom left panel (x = 0.0001) resembles the top left panel, with TE frequency moving both up and down.

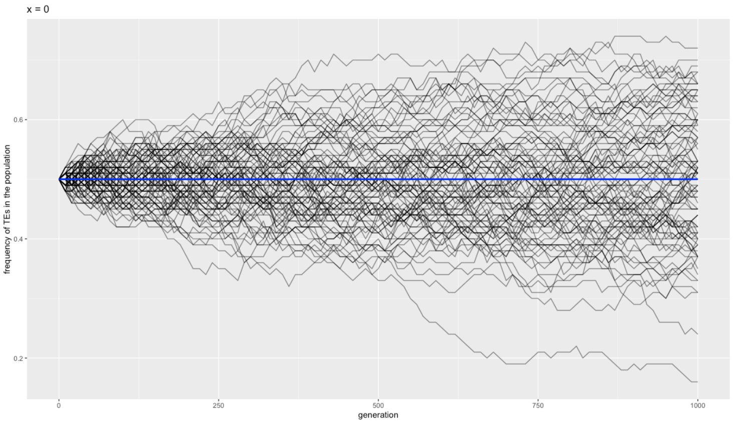

Figure S6.6: Validation of selection effects: Progression of TE population frequency through generations for x = 0. The blue line represents the average TE frequency.

Figure S6.6 focuses on the progression of TE population frequency for x = 0, with the blue line indicating the average TE frequency.

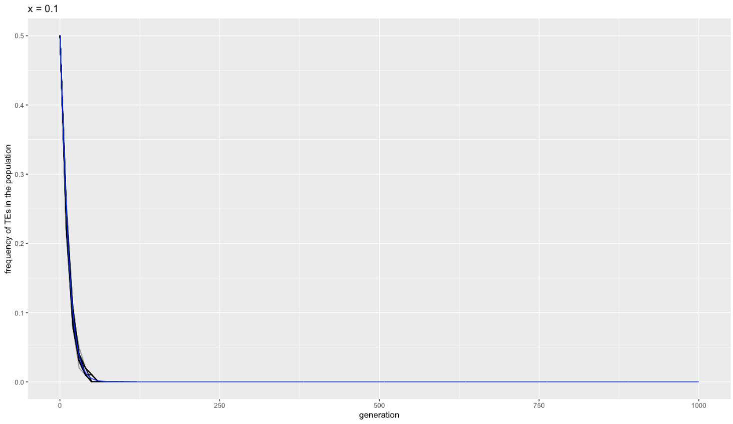

Figure S6.7: Validation of selection effects: Progression of TE population frequency through generations for x = 0.1. The blue line represents the average TE frequency.

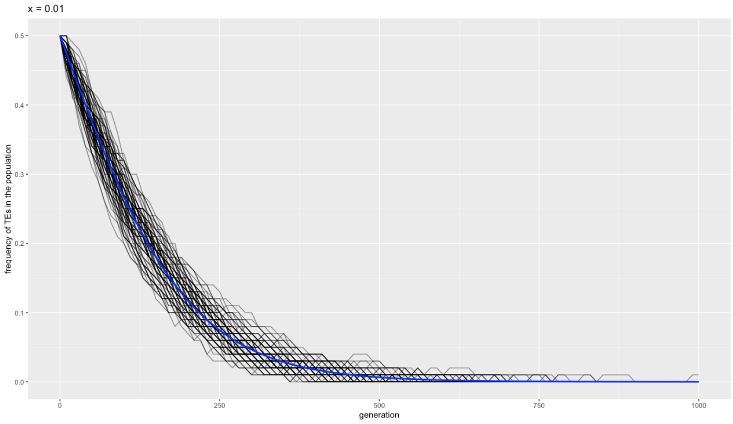

Figure S6.8: Validation of selection effects: Progression of TE population frequency through generations for x = 0.01. The blue line represents the average TE frequency.

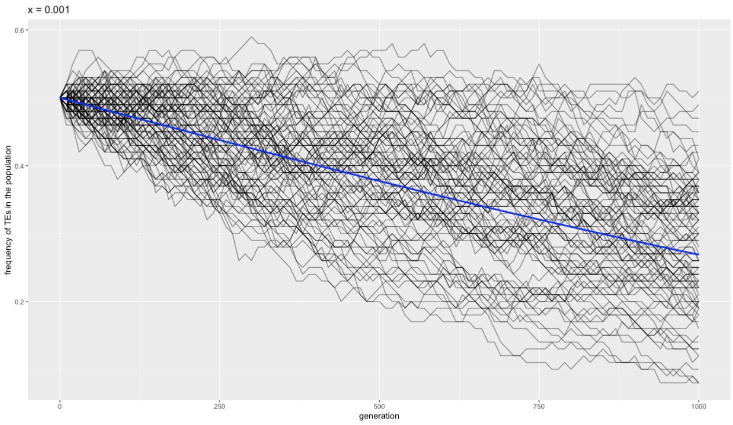

Figure S6.9: Validation of selection effects: Progression of TE population frequency through generations for x = 0.001. The blue line represents the average TE frequency.

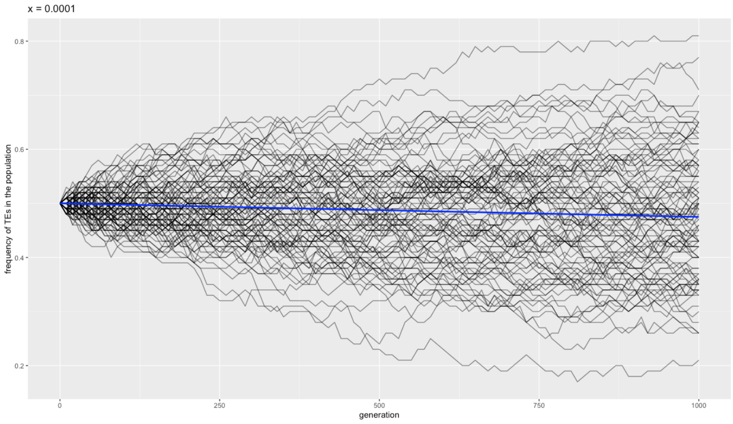

Figure S6.10: Validation of selection effects: Progression of TE population frequency through generations for x = 0.0001. The blue line represents the average TE frequency.

Figures S6.7, S6.8, S6.9, and S6.10 show the progression of TE population frequency through generations for selection coefficients x = 0.1, x = 0.01, x = 0.001, and x = 0.0001, respectively.

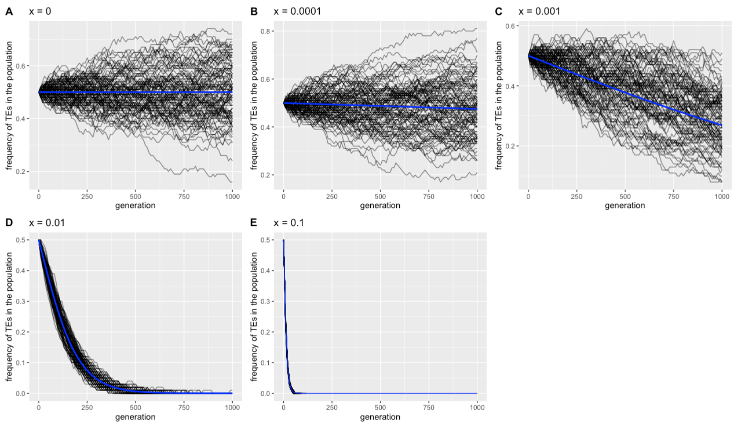

Figure S6.11: Validation of selection effects: Progression of TE population frequency through generations for different selection coefficients (x) on the same population, with average TE frequency represented by blue lines. Top left: x = 0, top right: x = 0.1, middle left: x = 0.01, middle right: x = 0.001, bottom: x = 0.0001.

Figure S6.11 is similar to Figure S6.5 but includes blue lines representing the average TE frequency for each selection coefficient.

S7 Insertion Validation

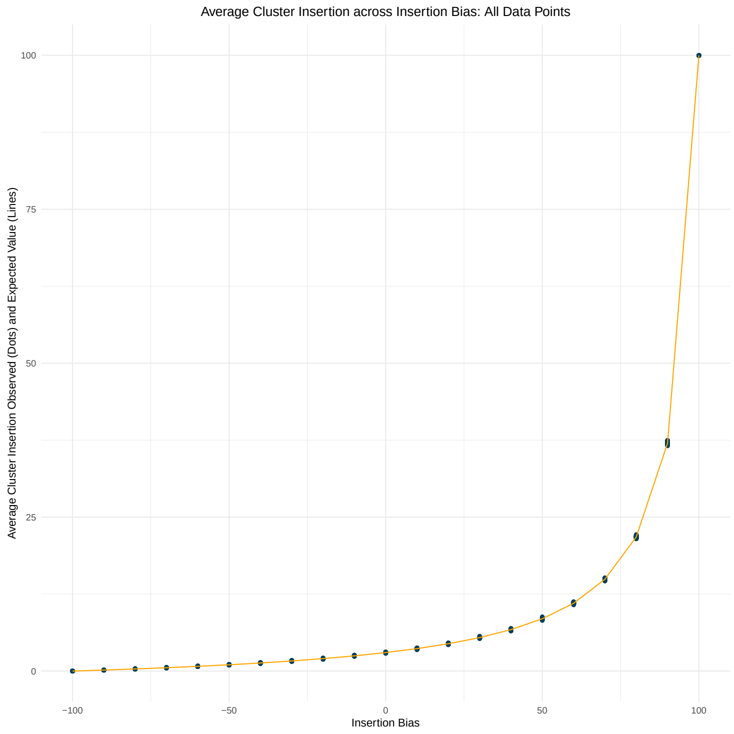

Figure S7.1: Validation of insertion: Average cluster insertion as a function of insertion bias. The x-axis represents the insertion bias ranging from -100 to 100 with a step size of 10. The y-axis shows the average cluster insertion. The dots in the plot represent the observed values, while the orange line represents the expected values. The observed and expected values match, showing the steep exponential curve.

This plot validates our implementation of insertion as piRNA cluster insertion is translating as expected with insertion bias.

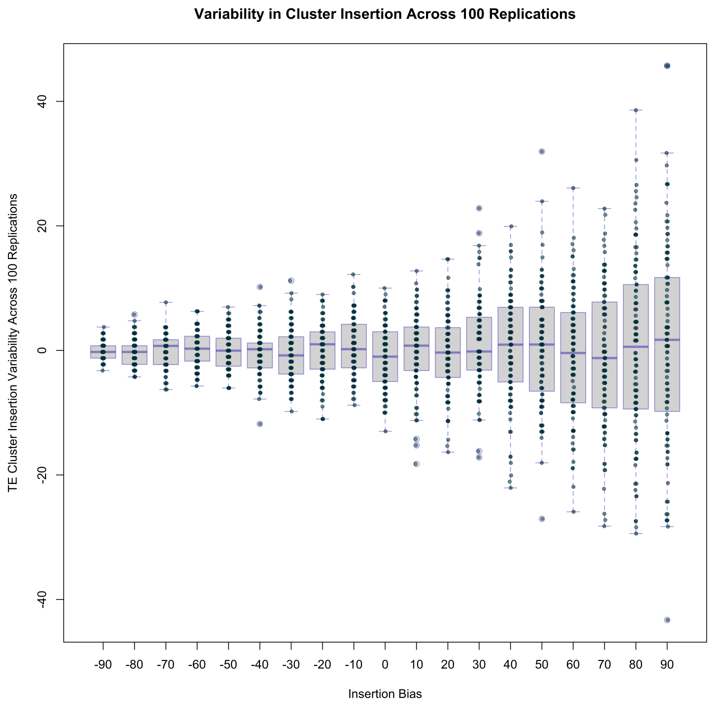

Figure S7.2: Validation of insertion: Variability in TE insertions across different levels of insertion bias, based on data from 100 replications. The y-axis represents the relative difference between 'avcli' and 'pc' columns, expressed as a percentage. The x-axis represents the categorical 'Insertion Bias' variable. Each boxplot summarizes the distribution of 'TE_insertions' for each level of 'Insertion Bias', indicating the median, interquartile range, and potential outliers.

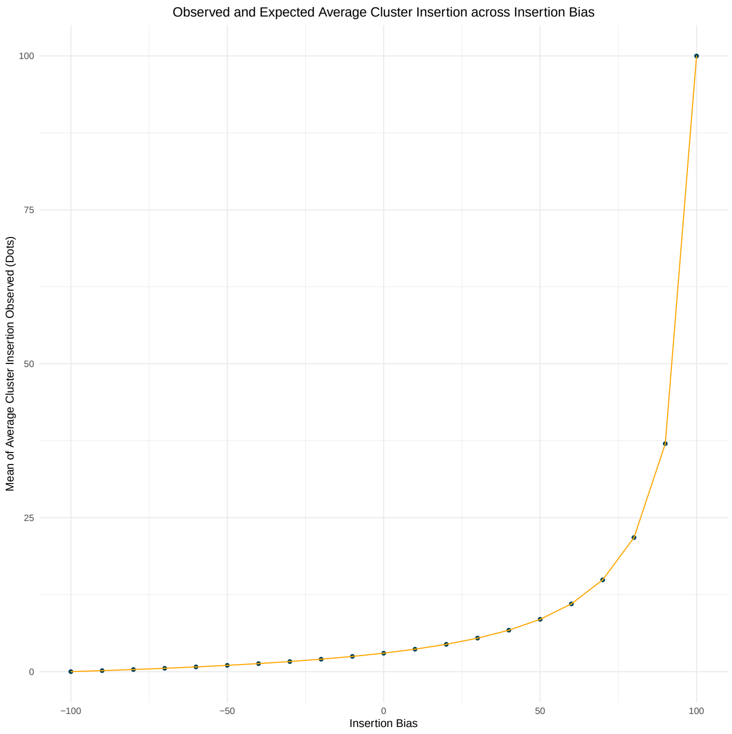

Figure S7.3: Validation of insertion: Mean of average cluster insertion across various insertion bias levels, with error bars indicating standard deviation. The orange line represents the expected values.

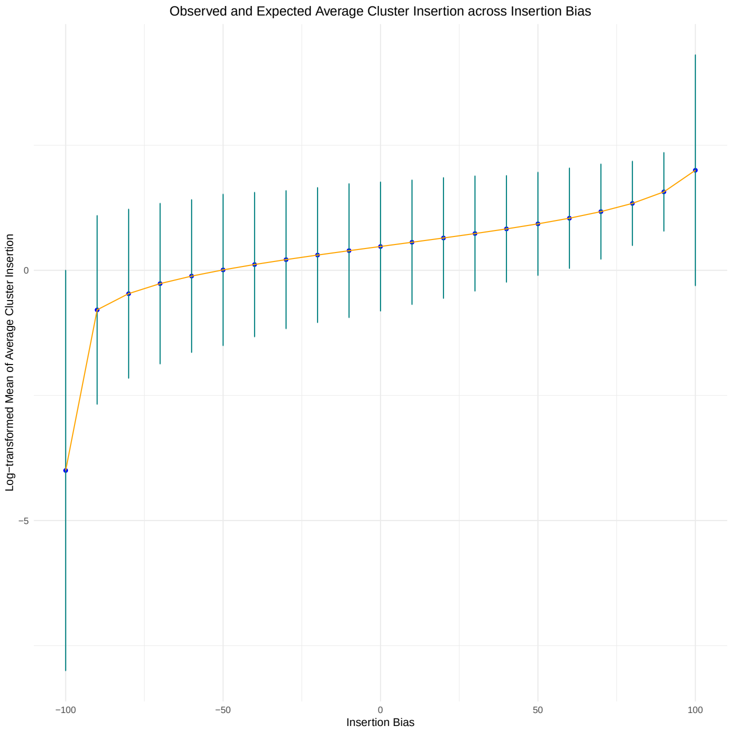

Figure S7.4: Validation of insertion: The same data as Figure S7.3, but with a logarithmic transformation applied to the y-values. The log scale helps to better visualize the variability in the data (shown by the error bars) and the deviation of the observed values (blue points) from the expected values (orange line).

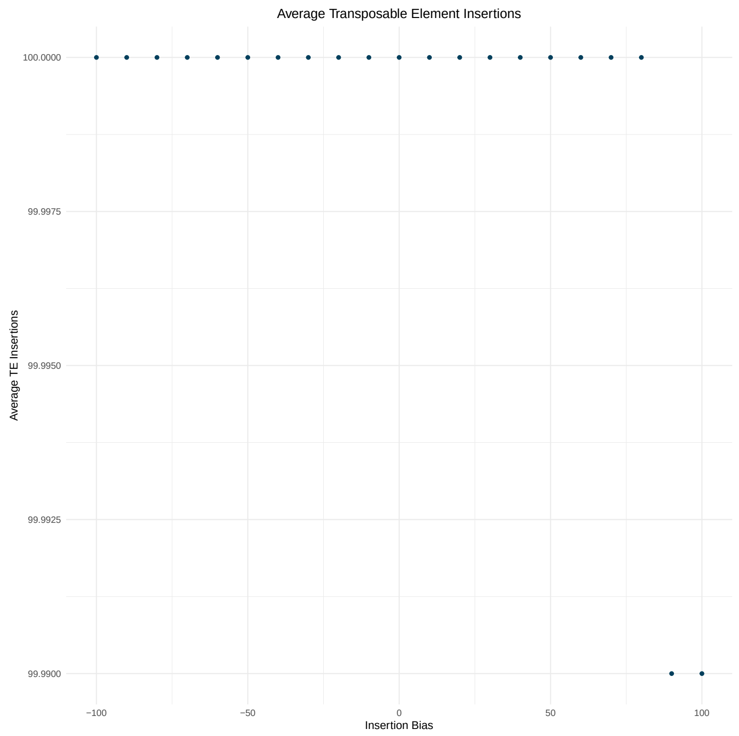

Figure S7.5: Validation of insertion: Average TE insertions (y-axis) and insertion bias (x-axis) for a single replicate. The x-axis represents the insertion bias varying from -100 to 100, while the y-axis depicts the average TE insertions for each bias level. Each data point represents the average TE insertions for a specific bias level for replicate 1.

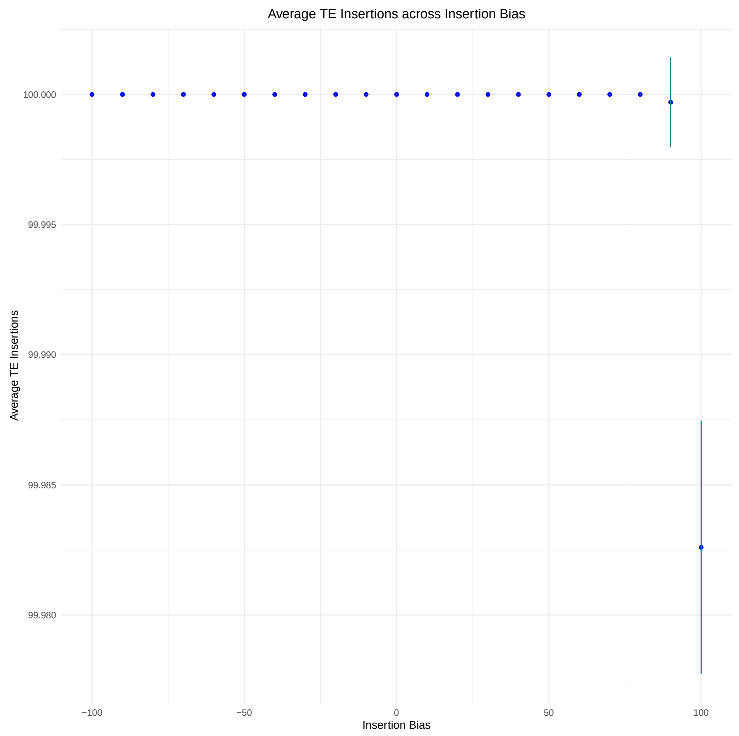

Figure S7.6: Validation of insertion: All replicates' mean average TE insertions (y-axis) against the insertion bias (x-axis). The plot includes error bars representing the standard deviation for the average TE insertions at each bias level. The x-axis represents the insertion bias, ranging from -100 to 100, while the y-axis depicts the mean of the average TE insertions for each bias level.

Across the 21 insertion bias values (sampleid 0 to 20), the mean of cli is calculated for all 100 replications, along with the standard deviation. The pc column represents the theoretical value of average cluster insertion for a given insertion bias with a 3% cluster size. The validation matches our expectations, confirming that the insertion is working as expected and the simulation successfully incorporates the user-defined TE insertions as specified.

S8 TE Competition with Direct Fitness Benefit

To explore the alternative hypothesis that cluster insertions might confer a direct fitness benefit, we extended our model. We introduced a beneficial fitness parameter, 'b', for each TE copy within a cluster, modifying the host fitness equation to $w = 1 - xn^t + bc$, where 'c' is the number of cluster insertions. We simulated four new scenarios in a complex genomic environment, varying both the strength of negative selection ('x') and the beneficial effect ('b') (Table 4).

The results, presented in Figure S8.1, show that a direct fitness benefit shifts the competitive outcome. With a small benefit and no negative selection (Figure S8.1A), neither TE type shows clear dominance. However, as the benefit of cluster insertion increases (Figure S8.1B) or is combined with negative selection

(Figure S8.1C), TEs with a higher insertion bias (blue) become increasingly dominant. When the benefit is strongest (Figure S8.1D), these self-regulating TEs almost completely out-compete their counterparts. This demonstrates that a direct fitness advantage for inserting into piRNA clusters provides a plausible evolutionary scenario where a higher insertion bias, that is, a form of self-regulation is favored.

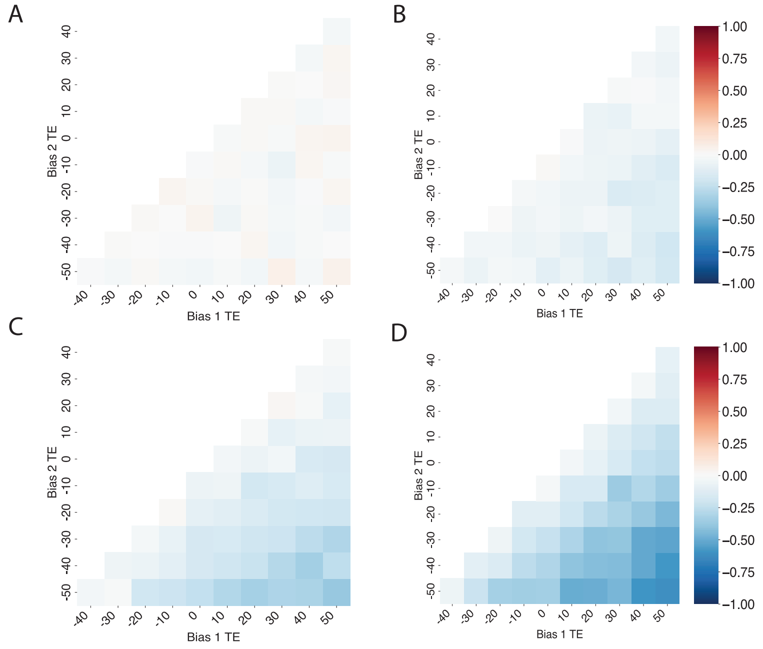

Figure S8.1: The impact of a direct fitness benefit for cluster insertion on TE competition. The four scenarios (A-D) correspond to the parameters detailed in Table 4, exploring how competition shifts as the negative selection pressure (x) and cluster insertion benefit (b) are varied. The color scale represents the competitive outcome: red indicates the dominance of TEs with a lower insertion bias (more selfish), while blue indicates the dominance of TEs with a higher insertion bias (more self-regulating). The scale is calculated same as in Figure 5 in the manuscript.

Part II

Supplementary Tables

This section presents the supplementary tables related to the validation steps described in the manuscript.

Table 1: Across the 21 insertion bias values (sampleid 0 to 20), the mean of cluster insertion is calculated for all 100 replications. The standard deviation is also calculated. We have another column named pc which is the theoretical value of average cluster insertion for a given insertion bias with a 3% cluster size.

sampleid	mean_cli	sd_meancli	pc	deviation_pc
-100	0.000	0.000	0.000	0.000
-90	0.162	0.0130	0.163	-0.000814
-80	0.341	0.0204	0.342	-0.00157
-70	0.542	0.0248	0.543	-0.000423
-60	0.769	0.0297	0.767	0.00154
-50	1.020	0.0307	1.020	-0.00111
-40	1.300	0.0360	1.310	-0.00454
-30	1.630	0.0417	1.640	-0.00397
-20	2.020	0.0448	2.020	-0.00160
-10	2.480	0.0459	2.470	0.00719
0	2.990	0.0514	3.000	-0.00930
10	3.640	0.0571	3.640	0.000316
20	4.430	0.0622	4.430	-0.00360
30	5.440	0.0707	5.430	0.00955
40	6.730	0.0861	6.730	0.00333
50	8.500	0.0932	8.490	0.00513
60	11.000	0.0994	11.000	-0.00867
70	14.900	0.112	14.900	-0.0104
80	21.800	0.144	21.800	0.00271
90	37.000	0.164	37.000	0.0114
100	100.000	0.00485	100.000	-0.0174

Table 2: Probability (p) that a TE jumps into a piRNA cluster depending on the insertion bias of the TE. The piRNA clusters accounts for 3% of the genome. Additionally, we show the number of expected insertions and observed with our software invadego for 1000 new TE insertions. Observed values are averaged over 1000 replicates. This demonstrates that our software correctly implements the insertion bias.

bias	p	expected	observed
100	1.000	1000	1000
90	0.370	370	369.72
80	0.218	218	217.65
70	0.149	149	149.66
60	0.110	110	109.57
50	0.085	85	84.91
40	0.067	67	67.16
30	0.054	54	54.42
20	0.044	44	44.36
10	0.036	36	36.68
0	0.030	30	30.11
-10	0.025	25	24.57
-20	0.020	20	20.19
-30	0.016	16	16.37
-40	0.013	13	12.98
-50	0.010	10	10.24
-60	0.008	8	7.65
-70	0.005	5	5.44
-80	0.003	3	3.48
-90	0.002	2	1.62
-100	0.000	0	0

Table 3: Supplementary Table 3: Statistical validation of TE establishment probability. For each insertion bias, the observed number of successful establishments out of 1000 simulation replicates was compared against a theoretical expectation of 81% using a two-sided binomial test. The 81% expectation is derived from the biological model of piRNA cluster maturation for a TE with no insertion bias, requiring it to survive approximately three generations to become established (0.9 * 0.9 = 0.81). This value serves as a baseline to test for deviations caused by insertion bias. The resulting p-values were adjusted for multiple comparisons using the Bonferroni correction. Significant deviation from the theoretical expectation is observed primarily at high positive insertion biases ($\geq +20$).

Insertion Bias	Successes	Replicates	Establishment Rate (%)	Adjusted p-value
-90	780	1000	78.0	0.33
-80	791	1000	79.1	> 0.99
-70	803	1000	80.3	> 0.99
-60	789	1000	78.9	> 0.99
-50	819	1000	81.9	> 0.99
-40	777	1000	77.7	0.17
-30	800	1000	80.0	> 0.99
-20	790	1000	79.0	> 0.99
-10	782	1000	78.2	0.50
0	797	1000	79.7	> 0.99
+10	779	1000	77.9	0.26
+20	756	1000	75.6	$5.0e-04$
+30	767	1000	76.7	$1.3e-02$
+40	758	1000	75.8	$8.7e-04$
+50	749	1000	74.9	$4.3e-05$
+60	731	1000	73.1	$2.4e-08$
+70	727	1000	72.7	$3.0e-09$
+80	619	1000	61.9	$1.4e-43$
+90	485	1000	48.5	$6.3e-116$

Table 4: Overview of TE Invasion Simulations with Cluster Insertion Benefit

**Scenario**	**Selection Parameters**	**Genome Structure**	**Premise and Rationale**
A	$x = 0$, $b = 0.01$	5 chr, 5 clusters	No negative selection pressure. Tests if a small direct benefit for cluster insertion is sufficient to favor self-regulating TEs.
B	$x = 0$, $b = 0.02$	5 chr, 5 clusters	No negative selection. Explores if doubling the direct benefit enhances the competitive advantage of self-regulating TEs.
C	$x = 0.01$, $b = 0.01$	5 chr, 5 clusters	Both pressures active. Models a trade-off between the harm of insertion (x) and the benefit (b).
D	$x = 0.01$, $b = 0.02$	5 chr, 5 clusters	Strongest regulatory incentive. Tests if a high benefit can decisively favor self-regulation even when TEs are deleterious.

Note: In all scenarios, the genomic and population parameters are held constant: N = 1000, initial TE population = 100, 100 replications, 500 generations, u = 0.1, 5 chromosomes, 5 piRNA clusters, and recombination rate 4cM/Mb. Selection parameters are the negative coefficient x and the cluster insertion benefit b (bcluins).
